# Neurostimulation for Advanced Parkinson Disease and Quality of Life at 5 Years: A Nonrandomized Controlled Trial

**DOI:** 10.1001/jamanetworkopen.2023.52177

**Published:** 2024-01-18

**Authors:** Stefanie T. Jost, Salima Aloui, Julian Evans, Keyoumars Ashkan, Anna Sauerbier, Alexandra Rizos, Jan Niklas Petry-Schmelzer, Alexandra Gronostay, Gereon R. Fink, Veerle Visser-Vandewalle, Angelo Antonini, Monty Silverdale, Lars Timmermann, Pablo Martinez-Martin, K. Ray Chaudhuri, Haidar S. Dafsari

**Affiliations:** 1 University of Cologne, Faculty of Medicine and University Hospital Cologne, Department of Neurology, Cologne, Germany; 2 Department of Neurology and Neurosurgery, Salford Royal NHS Foundation Trust, Manchester, UK; 3 Parkinson Foundation International Centre of Excellence, King’s College Hospital, London, UK; 4 Academic Health Science Centre, University of Manchester, Greater Manchester, UK; 5 Cognitive Neuroscience, Institute of Neuroscience and Medicine (INM-3), Research Centre Jülich, Jülich, Germany; 6 University of Cologne, Faculty of Medicine and University Hospital Cologne, Department of Stereotactic and Functional Neurosurgery, Cologne, Germany; 7 Department of Neurosciences (DNS), Padova University, Padova, Italy; 8 Department of Neurology, University Hospital Giessen and Marburg, Campus Marburg, Marburg, Germany; 9 Center for Networked Biomedical Research in Neurodegenerative Diseases (CIBERNED), Carlos III Institute of Health, Madrid, Spain; 10 Institute of Psychiatry, Psychology and Neuroscience, King’s College London, London, UK; 11 NIHR Mental Health Biomedical Research Centre and Dementia Biomedical Research Unit, South London and Maudsley NHS Foundation Trust and King’s College London, London, UK

## Abstract

**Importance:**

Deep brain stimulation of the subthalamic nucleus (STN-DBS) improves quality of life (QOL) in patients with advanced Parkinson disease (PD). However, controlled studies with more than 3 years of follow-up are lacking.

**Objective:**

To investigate the long-term effects of STN-DBS on QOL compared with standard-of-care medication (MED).

**Design, Setting, and Participants:**

In this prospective, observational, quasi-experimental, longitudinal nonrandomized controlled trial, 183 patients were screened for eligibility and 167 were enrolled from March 1, 2011, to May 31, 2017, at 3 European university centers. Propensity score matching for demographic and clinical characteristics was applied to 108 patients with PD (62 in the STN-DBS group and 46 in the MED group), resulting in a well-balanced, matched subcohort of 25 patients per group. Data analysis was performed from September 2022 to January 2023.

**Exposure:**

Treatment for PD of STN-DBS or MED.

**Main Outcomes and Measures:**

Assessments included Parkinson’s Disease Questionnaire 8 (PDQ-8), Unified PD Rating Scale–motor examination, Scales for Outcomes in PD–activities of daily living (ADL) and motor complications, and levodopa-equivalent daily dose. Within-group longitudinal outcome changes, between-group differences, and correlations of change scores were analyzed.

**Results:**

The study population in the analysis included 108 patients (mean [SD] age, 63.7 [8.3] years; 66 [61.1%] male). At 5-year follow-up, PDQ-8 and ADL worsened only in the MED group (PDQ-8 change, −10.9; 95% CI, −19.0 to −2.7; *P* = .01; ADL change: −2.0; 95% CI, −3.1 to −0.8; *P* = .002), whereas both outcomes remained stable in the STN-DBS group (PDQ-8 change, −4.3; 95% CI, −13.2 to 4.7; *P* = .34; ADL change, −0.8; 95% CI, −2.5 to 1.0; *P* = .38). Changes in PDQ-8 and ADL correlated moderately (*r*_s_ = .40, *P* = .008). Furthermore, STN-DBS outcomes were favorable for motor complications (median difference in change scores between STN-DBS and MED, −2.0; 95% CI, −4.0 to −1.0; *P* = .003), mobility (−1.0; 95% CI, −2.0 to 0; *P* = .03), and levodopa-equivalent daily dose reduction (−821.4; 95% CI, −1111.9 to −530.8; *P* < .001).

**Conclusions and Relevance:**

This study provides evidence of differences in QOL outcomes at 5-year follow-up between STN-DBS (stable) and MED (worsened), mainly driven by the favorable effect of STN-DBS on mobility (class IIb evidence). The association between changes in QOL and ADL, but not motor impairment or complications, highlights the relative importance of ADL outcomes for long-term DBS assessments.

**Trial Registration:**

German ClinicalTrials Registry: DRKS00006735

## Key Points

**Question:**

What are the long-term effects of deep brain stimulation of the subthalamic nucleus (STN-DBS) on quality of life (QOL), motor aspects, and medication requirements in patients with advanced Parkinson disease?

**Findings:**

This nonrandomized controlled trial of patients with advanced Parkinson disease found that at a 5-year follow-up QOL remained stable in the STN-DBS group and worsened in the standard-of-care medication group. The STN-DBS outcomes were favorable for mobility, motor complications, and levodopa-equivalent daily dose (class IIb evidence), and QOL and activities of daily living changes correlated moderately.

**Meaning:**

This trial found that patients who received STN-DBS had stable QOL at 5-year follow-up, primarily because of improved mobility, and highlights the importance of long-term improvement in outcomes related to activities of daily living.

## Teaser

This nonrandomized controlled trial of patients with advanced Parkinson disease examines the long-term effects of subthalamic nucleus deep brain stimulation, compared with standard-of-care medication, on quality of life.

## Introduction

Deep brain stimulation of the subthalamic nucleus (STN-DBS) is a well-established and efficacious treatment for improving quality of life (QOL), nonmotor symptoms, and motor symptoms in patients with advanced Parkinson disease (PD).^1,2,3^ Long-term studies with more than 5 years of follow-up have provided evidence of the beneficial effects of STN-DBS on motor complications,^4^ whereas conflicting results have been reported on long-term QOL outcomes up to 5 years after DBS surgery.^5,6^ A recent meta-analysis^7^ of studies of only patients undergoing STN-DBS reported a QOL improvement for up to 3 years after surgery, with subsequent decrements in these gains at a 5-year follow-up when QOL returns to preoperative status. In contrast, QOL worsens over time in patients treated with standard-of-care medication (MED), irrespective of patients’ PD stage.^8,9^ However, there is a lack of controlled studies comparing QOL outcomes of patients treated with STN-DBS and MED beyond a 36-month follow-up period.^10,11^ Therefore, we tested the hypotheses that in advanced PD at 5-year follow-up, QOL remains stable in patients undergoing STN-DBS and worsens in patients treated with MED and that this difference results in favorable outcomes of QOL and motor aspects as well as medication requirements in STN-DBS. Furthermore, we explored the association between changes in QOL and other clinical outcome parameters.

## Methods

### Study Design

We report the 1- and 5-year follow-up of the STN-DBS and MED arms of the ongoing, prospective, observational, controlled, multicenter Non-Motor International Longitudinal Study (NILS) study.^10,12,13,14,15^ Medical ethics committees of the participating centers approved the study protocol (Supplement 1). The study was performed under the Declaration of Helsinki.^16^ All patients provided written informed consent before participating in the study procedures. This report follows the Transparent Reporting of Evaluations With Nonrandomized Designs (TREND) reporting guideline for nonrandomized or quasi-experimental designs in the evaluation of interventions.^17^

### Participants

**Figure 1.**
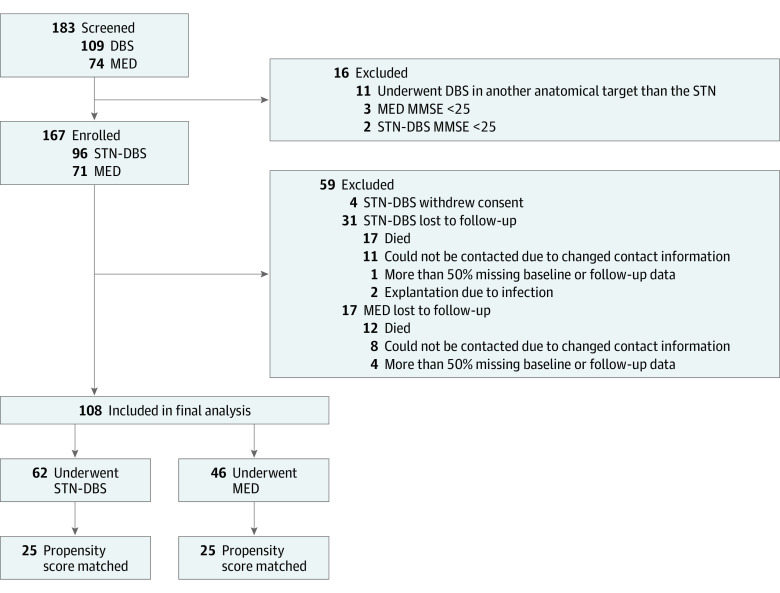
Patient Selection and Data Acquisition DBS indicates deep brain stimulation; MED, standard-of-care medical treatment; MMSE, Mini-Mental State Examination; STN-DBS, deep brain stimulation of the subthalamic nucleus.

Of 183 evaluable patients screened for eligibility, 167 were enrolled from March 1, 2011, through May 31, 2017, at 3 European university centers (Figure 1). Two patients switched between treatment arms and were therefore excluded from the final analysis (both after explantation of the pulse generator due to infection). The study population in the analysis included 108 patients, of whom 62 were in the STN-DBS and 46 in the MED group. Patients in the STN-DBS group were screened for surgery according to international guidelines.^18,19^ Indications for DBS were based on multidisciplinary assessments by movement disorders specialists, stereotactic neurosurgeons, neuropsychologists, and psychiatrists. Selection criteria for STN-DBS neurosurgery were (1) PD diagnosis based on the UK Brain Bank and Movement Disorders Society Clinical Diagnostic Criteria for PD^20,21^ and (2) absence of clinically relevant cognitive impairment, persistent severe psychiatric diseases, severe brain atrophy, or surgical contraindications.^22^ Surgical procedures for bilateral STN-DBS are described elsewhere and include visual targeting of the STN on preoperative magnetic resonance imaging and intraoperative refinement of targeting supported by microelectrode recordings.^23,24^ The MED group included only patients with advanced PD with dyskinesia, on/off fluctuations, or medication-refractory tremor to ensure comparability with patients undergoing STN-DBS. Patients in the MED group were considered candidates for DBS but, at that time, preferred nonsurgical standard-of-care pharmacotherapy^25^ for several reasons, including age, disease duration, medication requirements, or severity of motor and nonmotor symptoms. All patients received oral or transdermal pharmacologic treatment. Under the observational design of our study, patients in the STN-DBS group could switch off neurostimulation at any time during the study period, and patients in the MED group could decide to undergo DBS at any time. Patients who switched between these therapies were excluded from the current analyses. Moreover, the MED group served the specific purpose of providing a control group for an investigation of the long-term clinical efficacy of STN-DBS on QOL; therefore, we included only patients in the MED group for whom 5-year follow-up assessments of QOL were available in the NILS study at the time the database was closed for this analysis.

### Outcome Measures

At (preoperative) baseline, 1-year follow-up, and 5-year follow-up, outcome measures were assessed 30 to 60 minutes after dopaminergic medication intake on judgment of the patient and the movement disorders specialist that the optimal motor state was reached.^26,27^ This clinically defined medication-on state required at least 10 days of continuous intake of antiparkinsonian medication without medication changes.^28^ Patients in the STN-DBS group were postoperatively assessed in the clinically defined medication- and stimulation-on state with at least 10 days of continuous DBS without stimulation parameter changes and above-mentioned continuous medication intake.^26,27,28^

#### Primary Outcome

Quality of life was assessed with the Parkinson’s Disease Questionnaire 8 (PDQ-8), a short-form of the PDQ-39^29^ that covers 8 dimensions contributing to QOL (mobility, activities of daily living [ADL], emotional well-being, social support, cognition, communication, bodily discomfort, and stigma). The scale is recommended for QOL assessments by the Movement Disorders Society Scales Committee and is widely used in DBS studies.^30,31,32^ The PDQ-8 is reported as a Summary Index (PDQ-8 SI), ranging from 0 (no impairment) to 100 (maximum impairment).

#### Secondary Outcomes

Motor examination, ADL, and motor complications were assessed with the Scales for Outcomes in PD–motor scale (SCOPA-M). The SCOPA-M is a well-established, validated short version of the Unified Parkinson Disease Rating Scale (UPDRS)^33^ and highly correlates with the corresponding UPDRS parts.^34^ The SCOPA was chosen because its assessment time is approximately 4 times shorter than that of the UPDRS.^33,35^ The SCOPA subscales range from 0 (no impairment) to 42 (motor examination), 21 (ADL), and 12 (motor complications).^14^ On the basis of previously published conversion methods,^35,36^ we report motor examination as UPDRS III scores to help interpret the data. The levodopa equivalent daily dose (LEDD) was calculated according to the method by Jost et al.^37^ The total electrical energy delivered (TEED) was calculated using an established method by Koss et al,^38^ assuming a standard impedance of 1000Ω.^39,40^

### Adverse Events

Adverse events (AEs) were coded according to the Medical Dictionary for Regulatory Activities, version 26.0. The AEs were classified as mild, moderate, or severe or serious.^1^ Moreover, AEs were categorized as neurologic, psychiatric, and other.^41^

### Statistical Analysis

Statistical analyses were performed from September 2022 to January 2023 using SPSS Statistics, version 25.0 (IBM Corp). We applied propensity score matching for SPSS, version 3.04 to account for baseline differences between the STN-DBS and MED groups.^42^ Propensity score matching can be used to estimate treatment effects in observational studies when randomized treatment allocation is impossible.^43^ The following baseline demographic and clinical matching parameters were used: age, disease duration, LEDD, and SCOPA-M total score. We implemented nearest-neighbor matching (1:1 ratio) with a 0.3-SD caliper without replacement and conducted balance diagnostics based on the Cohen effect size (*d*<0.25).^44^

The assumption of normal distribution was tested with the Shapiro-Wilk test. Differences in baseline characteristics between the STN-DBS and MED group were analyzed using the χ^2^ test (dichotomous variables), Mann-Whitney *U* tests (continuous variables), or unpaired, 2-tailed *t* tests (when parametric criteria were fulfilled). Repeated-measures analysis of variance or Friedman tests were calculated to determine outcome changes within treatment groups between the 3 study visits (preoperative baseline, 1-year follow-up, and 5-year follow-up). Multiple comparisons attributable to multiple outcome parameters were corrected with the Benjamini-Hochberg procedure. A 2-sided *P* < .05 was the significance threshold. Post hoc, Wilcoxon signed-rank and unpaired, 2-tailed *t* tests were used to compare outcome changes between pairs of visits. Furthermore, Mann-Whitney *U* tests or unpaired, 2-tailed *t* tests of change scores from baseline to 5-year follow-up between the STN-DBS and MED groups (test_baseline_ − test_follow-up_) were calculated in a between-group analysis. We also explored post hoc PDQ-8 domains.

To determine the clinical relevance of the responses, we calculated relative changes analyses ([mean test_baseline_ − mean test_follow-up_]/mean test_baseline_), Cohen effect sizes for the within-group analyses ([mean test_baseline_ − mean test_follow-up_]/SD test_baseline_), and Cohen effect sizes for the between-group analysis according to a method by Morris^45^ for pretest-posttest-control group designs. Furthermore, we explored the association between changes in outcome measures using Spearman correlations.

## Results

### Baseline Characteristics of the Patients

A total of 108 patients (mean [SD] age, 63.7 [8.3] years; 66 [61.1%] male and 42 [38.9%] female) with a median (IQR) disease duration of 7.7 (5.5-13.0) years were included in the final analysis. Of these 108 patients from the original study cohort, at baseline, patients in the STN-DBS group had a significantly longer PD duration, worse QOL, more severe motor complications, and higher LEDD than patients in the MED group (Table 1). Propensity score matching resulted in a subcohort of 50 patients (25 patients in each group). Balance diagnostics indicated a good matching between the 2 groups (ie, no significant differences were found for the demographic and primary and secondary outcome parameters). The mean (SD) age of the matched cohort was 65.2 (9.1) years, and the median (IQR) disease duration was 7.4 (5.9-12.7) years. In the MED group, no patient underwent DBS or received any other device-aided treatment of PD during the 5-year follow-up period. In the STN-DBS group, no patient received any device-aided treatment of PD other than STN-DBS.

**Table 1.** Baseline Characteristics in the Original Cohort and Matched Subcohort

Characteristic	Original cohort (n = 108)						Matched subcohort (n = 50)					
	STN-DBS		MED		*P* value^a^	Δ (95% CI)^b^	STN-DBS		MED		*P* value^a^	Δ (95% CI)^b^
	No.	Mean (SD)	No.	Mean (SD)			No.	Mean (SD)	No.	Mean (SD)		
Age, y	62	62.6 (7.7)	46	65.2 (8.8)	.11	2.6 (−0.6 to 5.8)	25	64.8 (6.6)	25	65.5 (10.6)	.77	0.7 (−4.3 to 5.7)
Disease duration, median (IQR), y	62	10.4 (6.8 to 13.7)	46	5.7 (4.0 to 7.4)	<.001^c^	−4.5 (−6.2 to −2.8)	25	8.0 (6.1 to 13.0)	25	7.3 (5.5 to 12.5)	.37	−0.8 (−2.9 to 1.0)
Sex												
Female	62	25 (40.3)	46	17 (37.0)	.73	NA	25	9 (36)	25	10 (40.0)	.77	NA
Male	62	37 (59.7)	46	29 (63.0)			25	16 (64.0)	25	15 (60.0		
PDQ-8 Summary Index	60	31.8 (14.5)	46	21.3 (15.5)	<.001^c^	−10.5 (−16.3 to −4.7)	24	30.2 (14.8)	25	23.9 (15.3)	.15	−6.3 (−14.9 to 2.3)
UPDRS–motor examination	61	32.0 (12.8)	46	29.1 (13.2)	.25	−2.9 (−7.9 to 2.1)	25	32.5 (13.3)	25	31.2 (14.5)	.76	−1.3 (−9.3 to 6.6)
SCOPA-M total	61	22.8 (8.4)	46	19.5 (8.5)	.04^c^	−3.4 (−6.7 to −0.1)	25	21.9 (8.7)	25	21.0 (9.3)	.72	−0.9 (−6.0 to 4.2)
SCOPA-M Activities of daily living	61	7.1 (3.3)	46	6.3 (3.0)	.19	−0.8 (−2.1 to 0.4)	25	6.9 (3.6)	25	6.7 (3.1)	.80	−0.2 (−2.2 to 1.7)
SCOPA-M Motor complications, median (IQR)	61	4.0 (2.0 to 7.0)	46	2.0 (2.0 to 4.0)	.004^c^	−2.0 (−2.0 to 0.0)	25	3.0 (1.0 to 5.0)	25	3.0 (2.0 to 5.0)	.96	0.0 (−10 to 2.0)
LEDD	61	1153.6 (534.1)	46	694.7 (383.4)	<.001^c^	−459.0 (−643.9 to −283.0)	25	991.7 (447.2)	25	920.5 (344.5)	.53	−71.2 (−298.6 to 155.8)

Abbreviations: LEDD, levodopa equivalent daily dose; MED, standard-of-care medical treatment; NA, not applicable; PDQ-8, 8-item Parkinson’s Disease Questionnaire; SCOPA-M, Scales for Outcomes in Parkinson disease-motor scale; STN-DBS, subthalamic nucleus deep brain stimulation; UPDRS, Unified Parkinson Disease Rating Scale.

^a^ Mann-Whitney *U* tests or *t* tests when parametric test criteria were fulfilled.

^b^ Δ is the mean parameter_MED_ – mean parameter_STN-DBS_.

^c^ Statistically significant.

The results reported in this article relate to the matched cohort. Additionally, outcome changes of the original cohort are reported in eTables 1 and 2 in Supplement 2. Furthermore, in eTable 3 in Supplement 2, effect sizes at 1-year follow-up of the matched cohort are presented.

The time from (preoperative) baseline to follow-up assessments did not differ significantly between the matched groups. The median (IQR) time from preoperative baseline to surgery was 1 (1-3) day(s).

### Quality of Life

**Figure 2.**
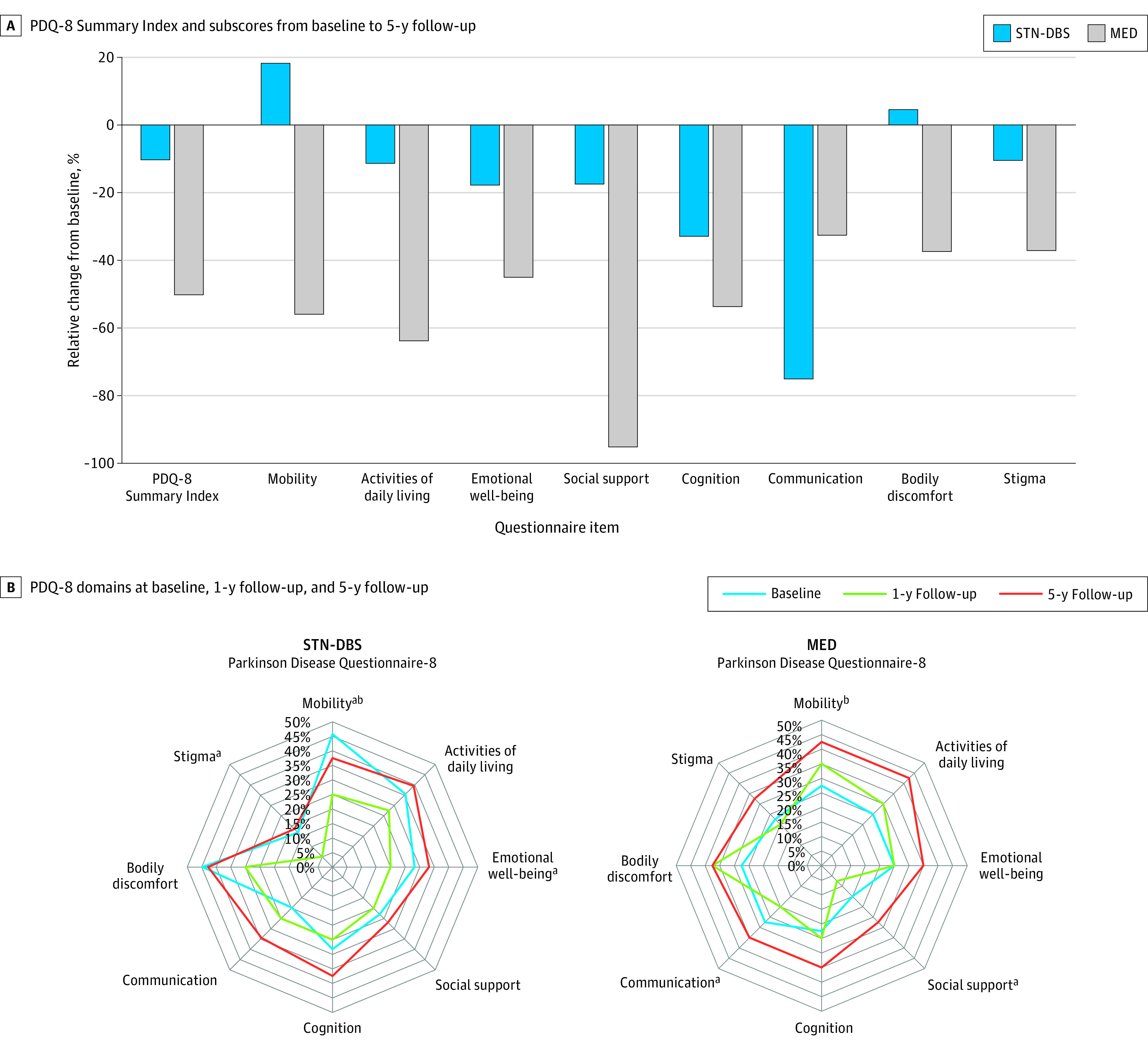
Domains of Quality of Life and Motor Aspects in the Patients Receiving Deep Brain Stimulation of the Subthalamic Nucleus (STN-DBS) vs Standard-of-Care Medical Treatment (MED) For the Parkinson’s Disease Questionnaire 8 (PDQ-8), positive scores indicate improvement and negative scores indicate worsening. The PDQ-8 domain scores are illustrated as the percentage of maximum scores. More extensive areas represent more severe impairment. ^a^Significant longitudinal within-group changes among the 3 visits. ^b^Significant between-group differences at 5-year follow-up (STN-DBS vs MED).

The primary outcome (PDQ-8 SI) worsened significantly from baseline to 5-year follow-up by 49.4% in the MED group (PDQ-8 SI change, −10.9; 95% CI, −19.0 to −2.7; *P* = .01), whereas it remained stable in the STN-DBS group (PDQ-8 SI change, −4.3; 95% CI, −13.2 to 4.7; *P* = .34) (Table 2 and Table 3) The between-group difference in the mean change from baseline to 5-year follow-up was 6.6 points, and the effect size of this difference was moderate. In the STN-DBS group, the PDQ-8 SI improvement from baseline was significant at the 1-year follow-up but due to detriments in gains was not significant at the 5-year follow-up. In the MED group, the PDQ-8 SI remained stable from baseline to 1-year follow-up and significantly worsened after that. The PDQ mobility domain outcome from baseline to 5-year follow-up was favorable in the STN-DBS compared with the MED group (median difference in change scores between STN-DBS and MED −1.0; 95% CI, −2.0 to 0; *P* = .03) (Figure 2A and B).

**Table 2.** Outcomes at Baseline, 1-Year Follow-Up, and 5-Year Follow-Up in the Matched Cohort

Outcome	STN-DBS											MED												
	Baseline		1-y FU		5-y FU		Baseline vs 1-y FU^a^		Baseline vs 5-y FU^a^		Baseline vs 1-YFU vs 5-YFU, *P* value^b^	Baseline		1-y FU		5-y FU			Baseline vs 1-y FU^a^		Baseline vs 5-y FU^a^		Baseline vs 1-y vs 5-y FU, *P* value^b^	STN-DBS vs MED, Δ (95% CI)^c^
	No.	Mean (SD)	No.	Mean (SD)	No.	Mean (SD)	*P* value	Δ (95% CI)	*P* value	Δ (95% CI)		No.	Mean (SD)	No.	Mean (SD)	No.	Mean (SD)	*P* value	Δ (95% CI)	*P* value	Δ (95% CI)	*P* value		
PDQ-8 Summary Index	24	30.2 (14.8)	25	22.5 (16.2)	23	33.3 (18.2)	.01^d^	7.3 (1.6 to 13.0)	.34	−4.3 (−13.2 to 4.7)	.02^d^	25	23.9 (15.3)	23	25.1 (14.9)	23	35.7 (18.4)	.71	−1.0 (−6.1 to 4.2)	.02^d^	−10.9 (−19.0 to −2.7)	.01^d^	.26	−6.6 (−18.4 to 5.1)
UPDRS–motor examination	25	32.5 (13.3)	23	30.1 (11.6)	17	32.5 (14.3)	.11	3.7 (−0.9 to 8.2)	.43	2.0 (−3.2 to 7.2)	.14	25	31.2 (14.5)	23	30.2 (13.9)	24	35.5 (15.7)	.71	0.9 (−4.2 to 6.1)	.16	−4.0 (−9.7 to 1.7)	.21	.13	−6.0 (−13.9 to 1.9)
SCOPA-M total	25	21.9 (8.7)	23	17.7 (8.7)	17	20.5 (10.8)	.003^d^	4.8 (1.8 to 7.8)	.20	1.9 (−1.1 to 5.0)	.009^d^	25	21.0 (9.3)	23	19.0 (9.1)	23	25.1 (9.9)	.31	1-6 (−1.6 to 4.8)	.008^d^	−4.5 (−7.4 to −1.6)	.002^d^	.003^d^	−6.4 (−10.6 to −2.3)
SCOPA-M activities of daily living	25	6.9 (3.6)	25	5.1 (3.5)	25	7.7 (4.2)	.02^d^	1.8 (0.3 to 3.4)	.38	−0.8 (−2.5 to 1.0)	.009^d^	25	6.7 (3.1)	23	6.3 (3.5)	23	8.4 (3.7)	.66	0.2 (−0.8 to 1.2)	.006^d^	−2.0 (−3.1 to −0.8)	.002^d^	.25	−1.2 (−3.3 to 0.9)
SCOPA-M motor Complications, median (IQR)	25	3.0 (1.0 to 5.0)	25	0.0 (0.0 to 2.0)	25	2.0 (0.0 to 3.0)	.001^d^	−1.5 (−2.5 to 0.0)	.01^d^	−1.5 (−2.5 to 0.0)	.009^d^	25	(3.0) (2.0 to 5.0)	24	(2.0) (1.0 to 3.0)	24	(4.0) (2.5 to 5.0)	.04^d^	−1.0 (−2.0 to 0.0)	.04^d^	1.0 (0.0 to 2.0)	.002^d^	.003^d^	−2.0 (−4.0 to −1.0)
LEDD	25	991.7 (447.2)	25	374.7 (260.4)	24	525.4 (395.3)	<.001^d^	617.0 (417.6 to 816.3)	<.001^d^	480.5 (247.4 to 713.5)	<.001^d^	25	920.5 (344.5)	22	1077.2 (416.2)	25	1034.0 (597.0)	.01^d^	−188.8 (−333.8 to −43.8)	<.001^d^	−340.9 (−521.7 to −160.1)	<.001^d^	<.001^d^	−821.4 (−1111.9 to −530.8)

Abbreviations: FU, follow-up; LEDD, levodopa equivalent daily dose; MED, standard-of-care medical treatment; PDQ-8, Parkinson Disease Questionnaire 8; SCOPA-M, Scales for Outcomes in Parkinson Disease–motor scale; STN-DBS, subthalamic nucleus deep brain stimulation; UPDRS, Unified Parkinson Disease Rating Scale.

^a^ Wilcoxon signed-rank test or paired *t* tests between baseline and follow-up to analyze within-group changes of outcome parameters.

^b^ Friedman test or repeated-measures analysis of variance when parametric test criteria were fulfilled.

^c^ Mann-Whitney *U* tests or repeated-measures analysis of variance were used to explore between-group differences of change scores (baseline vs 5-year follow-up) between the STN-DBS and MED group. Δ is the mean parameter_MED_ – mean parameter_STN-DBS_.

^d^ Statistically significant.

**Table 3.** Relative Changes and Effect Sizes for the Matched Cohort^a^

Outcome	Within-group changes (baseline to 5-y follow-up)				
	Relative change, %		Effect size (classification)		Between-group differences (classification), effect size (favoring STN-DBS)
	STN-DBS	MED	STN-DBS	MED	
PDQ-8 Summary Index	−10.3	−49.4	0.21 (small)	0.77 (moderate)	0.57 (moderate)
UPDRS–motor examination	0.2	−13.8	0.01 (negligible)	0.30 (small)	0.31 (small)
SCOPA-M total score	6.4	−19.5	0.16 (negligible)	0.44 (small)	0.60 (moderate)
SCOPA-M activities of daily living	−11.6	−25.4	0.22 (small)	0.55 (moderate)	0.26 (small)
SCOPA-M motor complications score	47.1	−27.3	0.59 (moderate)	0.56 (moderate)	1.11 (large)
LEDD	47.0	−35.4	1.04 (large)	0.33 (small)	1.95 (large)

Abbreviations: LEDD, levodopa equivalent daily dose; MED, standard-of-care medical treatment; PDQ-8, Parkinson Disease Questionnaire 8; SCOPA-M, Scales for Outcomes in Parkinson Disease–motor scale; STN-DBS, subthalamic nucleus deep brain stimulation; UPDRS, Unified Parkinson Disease Rating Scale.

^a^ Relative change was calculated as (mean test_baseline_ − mean test_follow-up_)/mean test_baseline_ × 100. The Cohen effect size was calculated as (mean test_baseline_ − mean test_follow-up_)/SD test_baseline_. The Cohen effect size for differences in change scores between the STN-DBS and MED group was calculated as (mean pre-post change_treatment group_ − mean pre-post change_control group_)/SD pretest_pooled groups_. The Cohen *d* can be classified as small (0.20-0.50), moderate (0.50-0.80), or large (≥0.80).

### Motor Outcomes, Medication Requirements, TEED, and AEs

The SCOPA-M total score worsened significantly from baseline to 5-year follow-up by 19.5% in the MED group (SCOPA-M total change, −4.5; 95% CI, −7.4 to −1.6; *P* = .008), whereas a 6.4% improvement in the STN-DBS group was statistically insignificant (SCOPA-M total change, 1.9; 95% CI, −1.1 to 5.0; *P* = .20). The between-group SCOPA-M total outcome difference was 6.4 points in favor of STN-DBS (SCOPA-M total outcome difference, −6.4; 95% CI, −10.6 to −2.3; *P* = .003), and this difference’s effect size was moderate. In the MED group, the worsening of the SCOPA-M total score resulted from a 25.4% worsening of ADL (SCOPA-M ADL change, −2.0; 95% CI, −3.1 to −0.8; *P* = .006) and a 27.3% worsening of motor complications (SCOPA-M motor complications change, 1.0; IQR, 0.0-2.0; *P* = .04). In the STN-DBS group, the improvement of SCOPA-M total score was driven by a 47.1% improvement of motor complications (SCOPA-M motor complication change, −1.5; IQR, −2.5 to 0.0; *P* = .01). From baseline to 5-year follow-up, LEDD increased by 17.0% in the MED group 1 (LEDD change, −340.9; 95% CI, −521.7 to −160.1; *P* < .001) and decreased by 62.2% in the STN-DBS group (LEDD change, 617.0; 95% CI, 417.6 to 816.3; *P* < .001).

The mean (SD) TEED was 53.4 (34.1) μJ/s at 1-year follow-up (57.7 [50.5] μJ/s for the left and 49.1 [39.5] μJ/s for the right hemisphere) and 102.1 (67.6) μJ/s at 5-year follow-up (108.5 [87.5] μJ/s for the left and 95.7 [77.5] μJ/s for the right hemisphere). The TEED increased by 91.0% from 1- to 5-year follow-up (*P* < .001).

In the STN-DBS group, we observed 39 serious AEs in 17 patients, which all resolved without major sequelae with 1 exception unrelated to PD in 1 patient, who underwent surgical colostomy for ileus. Of these serious AEs, 13 were categorized as surgical or device related (all of which were not life-threatening and resolved without sequelae), 8 as neurologic, 7 as psychiatric, and 11 as other reasons. Furthermore, we observed 360 AEs (262 mild, 84 moderate, and 14 severe), of which 283 AEs in 25 patients were categorized as neurologic, 47 AEs in 20 patients as psychiatric, and 36 AEs in 17 patients resulted from other categories. In our cohort, we observed 2.33 neurologic AEs per year, 0.43 psychiatric AEs per year, 0.10 surgical and device-related AEs per year, and 0.38 other AEs per year.

### Spearman Correlation Analyses

There was a significant correlation of moderate strength between change scores of PDQ-8 SI and SCOPA-M ADL (*r*_s_ = 0.40, *P* = .008) and a weak correlation between change scores of PDQ-8 mobility and SCOPA-M ADL (*r*_s_ = 0.35, *P* = .02). We found no evidence that changes in PDQ-8 SI and UPDRS III, SCOPA-M motor complications, LEDD, or TEED were correlated.

## Discussion

### Quality of Life

The main finding of this prospective, quasi-experimental, nonrandomized controlled trial is that QOL outcomes at 5-year follow-up were stable in the STN-DBS group and worsened in the MED group. To our knowledge, this is the first report of 5-year QOL outcomes following STN-DBS for patients with advanced PD compared with patients receiving only standard-of-care medical treatment. The between-group difference in the mean change of PDQ-8 SI from baseline to 5-year follow-up of 6.6 points favoring STN-DBS is a clinically relevant finding^46^ with a moderate effect size. The clinically relevant difference of QoL outcome at 5-year follow-up observed in our advanced PD cohort was not reported in patients undergoing STN-DBS for very early PD without motor complications or dyskinesia.^47^ The favorable effect of STN-DBS on overall QoL was mainly driven by the mobility domain, for which we observed a moderate effect size of QoL outcome difference (class IIb evidence). Furthermore, between-group analyses were favorable for STN-DBS for other QoL aspects, such as ADL, social support, bodily discomfort, and stigma (all small effect size), highlighting the advantages of neurostimulation in treating PD motor symptoms. In contrast, the communication domain outcome was favorable in the MED group (small effect size), which aligns with a previous study,^48^ which reported a worsening of speech intelligibility as a common AE of subthalamic stimulation.

Furthermore, correlation analyses showed the association between QOL and ADL change scores but not motor examination or complications. This finding confirms the findings of an earlier study^49^ and highlights the relative importance of ADL outcomes for evaluating the long-term clinical efficacy of DBS for PD. Future research should identify preoperative factors associated with an unfavorable long-term QOL outcome of STN-DBS.

### Motor Outcomes, Medication Requirements, TEED, and AEs

To our knowledge, this is the first report of class IIb evidence of beneficial long-term effects of STN-DBS on motor complications and medication requirements at a 5-year follow-up. In line with the literature,^50^ in the STN-DBS group, we observed a marked improvement of motor fluctuations from baseline to 1- and 5-year follow-up. In the MED group, the improved motor complications at the 1-year follow-up most likely resulted from optimizing patients’ medical regimens. The subsequent worsening of dyskinesia from 1- to 5-year follow-up, accompanied by a rebound of motor fluctuations to baseline levels, most likely reflects disease progression and possibly motor complications attributable to long-term dopaminergic therapy in advanced PD. As expected, these factors contributed to a favorable outcome of motor complications in STN-DBS compared with MED at the 5-year follow-up.

Changes in medication requirements differed between the STN-DBS and MED groups. The 42% LEDD reduction after STN-DBS at 5-year follow-up was well within the range reported in the literature.^6,49,51^ Medication requirements in the MED group increased significantly during the 5-year study.

The 91% TEED increase from 1- to 5-year follow-up in our cohort was smaller than the 320% increase from 6-month to 5-year follow-up observed in patients without preoperative motor fluctuations.^47^ The greater TEED increase in the study by Hacker et al^47^ likely resulted from the relatively low TEED at a 6-month follow-up, at which patients were still at a very early PD stage. On the basis of the sum of patient-years after undergoing DBS, we observed more AEs than reported in retrospective studies with long-term follow-up periods (our cohort: 2.33 neurologic AEs per year, 0.43 psychiatric AEs per year, 0.10 surgical and device-related AEs per year, and 0.38 other AEs per year; Hamburg cohort: 0.33 neurologic AEs per year, 0.13 psychiatric AEs per year, 0.04 surgery and device-related AEs per year, and 0.24 other AEs per year), ^41^ possibly because our patients were monitored more closely in a prospective study. The number of AEs per year in our study was considerable and has been reported in similar dimensions in studies with shorter follow-up periods.^1,2^ The relevance of the number of AEs per year is that they demonstrate the importance of thorough preoperative assessments of risk-benefit ratios. Consequently, in the clinical context, the preoperative impairments of QOL, social, functional, motor, and nonmotor status must provide the potential for clinically relevant improvements.

### Limitations

This study has some limitations. Because this was a multicenter, prospective, observational, but nonrandomized trial, we used a propensity score analysis to reduce confounding bias. In our study, a randomized trial of the long-term effects of DBS would have withheld an efficacious and safe treatment from severely impaired patients with advanced PD for 5 years. In such clinical scenarios, in which randomized trials are unethical, not feasible, or too costly from a health expenses or study conductance perspective, propensity score matching provides a tool to precisely match the baseline characteristics of statistical twins in treatment arms, which creates a quasi-experimental study design and increases the possibility of causal inference.^52^ Propensity score matching has been used for this specific purpose in studies on DBS for PD^11,12,53^ and in other neurologic diseases, such as multiple sclerosis.^54^ However, it can only be applied to those parameters that are known and measured. In our study, this did not account for other possibly relevant parameters (eg, impulsive and compulsive behavior or the severity of impairments in specific cognitive domains).^55,56,57^ Therefore, propensity score matching cannot replace randomized trials. Moreover, propensity score matching requires a significant overlap between characteristics of individuals exposed and unexposed to a specific treatment; otherwise, the matched sample is not large or representative enough. We chose 0.3-SD caliper width as the best possible solution to provide a sufficient number of matched pairs for statistical analyses and still minimize potential bias.^58^ A smaller caliper width would have resulted in a smaller matched cohort, which would have limited the statistical power and the interpretation of our results. In this context, we acknowledge that the cohort size of our study is rather small (108 in the original cohort and particularly 50 in the matched cohort). Nonetheless, considering a 5-year follow-up period, this was one of the biggest cohorts in studies of its kind. Although balance diagnostics^44^ indicated that the matching process led to balanced baseline characteristics between the STN-DBS and MED groups, statistically insignificant baseline differences remained, which may have influenced the between-group differences of outcomes. In this context, we acknowledge that previous studies have shown that higher baseline QOL impairment predicts greater postoperative QOL improvement in a follow-up period up to 3 years.^59,60^

Another important limitation of our study is that the preoperative PDQ-8 SI as the main outcome parameter was higher at baseline in the STN-DBS group. This baseline difference was not significant, possibly because of the small size of the matched groups. However, it is unlikely that the favorable effects of STN-DBS on QOL resulted from regression to the mean, because we observed differential effects of both treatments on specific aspects of QOL. In the STN-DBS group, QOL aspects related to motor symptoms were favorable, whereas the communication outcome was unfavorable.

Because we were interested in the real-life long-term effects of STN-DBS, we did not conduct a motor examination in the medication-off state, which is one of the main limitations of our study. To further elucidate the differential effects of STN-DBS on specific QOL aspects related to motor, cognitive, and speech outcomes, further studies with blinded assessments in the medication- and stimulation-off and -on states are needed. In this context, another limitation of our study is the lack of long-term follow-up motor examination data for some patients, as assessments could only take place via telephone during the COVID-19 pandemic. Furthermore, one has to acknowledge that dropouts from long-term studies occur more frequently among poor-performing and more severely impaired patients (eg, nursing home residents).^6^ Indeed, patients in nursing homes are often difficult to follow up in DBS clinics because in the later stages of the disease patients may already be too disabled to go to the hospital. However, the dropout rates in our study were well within the range of previous work.^61^ The proportion of patients who died during the 5-year follow-up period was similar in the STN-DBS arm and in the MED arm. Furthermore, we included only QOL assessments provided by patients themselves and not by their caregivers, which may provide further information in more advanced PD stages.

## Conclusions

This nonrandomized controlled study provides evidence of differences in QOL outcomes at 5-year follow-up between STN-DBS (stable) and MED (worsened) groups, mainly driven by the favorable effect of STN-DBS on mobility (class IIb evidence). The association between changes in QOL and ADL, but not motor impairment or complications, highlights the relative importance of ADL outcomes for long-term DBS assessments. These findings may provide helpful information when counseling patients on the efficacy of STN-DBS for PD and monitoring patients postoperatively in long-term follow-up.

## Notes

Supplement 1.
English Translation of the IRB Protocol

Supplement 2. **eTable 1.** Outcomes at Baseline, 1-Year Follow-Up, and 5-Year Follow-Up in the Original Cohort **eTable 2.** Relative Changes and Effect Sizes for the Original Cohort at 1-Year and 5-Year Follow-Up **eTable 3.** Relative Changes and Effect Sizes for the Matched Cohort at 1-Year and 5-Year Follow-Up **eFigure.** Domains of Quality of Life and Motor Aspects in the Matched Cohort at Baseline, 1-, and 5-Year Follow-Up

Supplement 3.
Nonauthor Collaborators

Supplement 4.
Data Sharing Statement
