# Supplementary material for: Neurostimulation for Advanced Parkinson Disease and Quality of Life at 5 Years: A Nonrandomized Controlled Trial
Source: JAMA Netw Open. 2024 Jan 18;7(1):e2352177. doi: 10.1001/jamanetworkopen.2023.52177 (PMC10797423; doi:10.1001/jamanetworkopen.2023.52177)
Supplement: Supplement 1. — English Translation of the IRB Protocol [file jamanetwopen-e2352177-s001.pdf]

# Application for the Approval of the Ethics Committee of the Medical Faculty of the University of Cologne

## A. Formalities

### 1. Title of the project

REAL-DBS-PD: **Re**gistry for **A**pomorphine and **L**-Dopa Infusion Therapies and **D**eep **B**rain **S**timulation in Patients with **P**arkinson's **D**isease
(German title: „Register zur Behandlungspraxis mit Apomorphin-, L-Dopa- Infusionstherapie und Tiefe Hirnstimulation bei Patienten mit Idiopathischem Parkinson-Syndrom“)

### 2. Applicant

Dr. Haidar Salimi Dafsari, MD
Clinical Research Fellow
Department of Neurology
University Hospital Cologne, Kerpener Str. 62, 50924 Cologne
Tel: + 49- (0) 221-478-4015
Fax: + 49- (0) 221-478-97819
Email: haidar.dafsari@uk-koeln.de

### 3. Type and number of study centers

Department of Neurology, University of Cologne

### 4. Has an application been submitted to another ethics committee regarding the same project?

No.

### 5. Written declaration of consent by the director of the department

I hereby declare my approval for this study to be conducted at the Department of Neurology, University of Cologne: "REAL-DBS-PD: Registry for Apomorphine and L-Dopa Infusion Therapies and Deep Brain Stimulation in Patients with Parkinson's Disease".

_______________
Prof. Dr. G.R. Fink

### 6. Information on the extent to which the study is financed externally

Not applicable.

**7. Description of study-related additional health-costs, finance plan**
Not applicable.

**8. Written declaration of consent for charging of consulting fees in case of industry sponsored studies**
Not applicable.

**9. In multicenter studies: number of patients to be included in the study in Cologne**
500-600

**10. Declaration: "I hereby declare to the ethics committee of the medical institutions of the University of Cologne that I guarantee the requirements (material, personnel, and organizational) of the clinical trial."**

**11. In case of use of radioactive substances or ionizing radiation in humans: Indicate in the application / test plan, to what extent, when using ionizing radiation (also with conventional methods) these are carried out for sole research purposes or based on a clinical indication (paragraph 23 and 80 StrlSchV). As a precaution, the ethics committee points out the fact that, in case the primary purpose is not for a clinical indication, an authorization has to be issued by the Federal Office for Radiation Protection in Salzgitter.**
Not applicable.

## B. Study description

**12. Scientific objective**
Parkinson's disease (PD) is a neurodegenerative disease affecting approximately 180,000 patients in Germany. It is characterized by the cardinal symptoms bradykinesia, rigidity, tremor, and postural instability. These symptoms are the result of a loss of dopaminergic neurons in the area of the substantia nigra pars compacta (SNc).
In addition to these motor symptoms, the disease pattern is also characterized by a variety of non-motor symptoms (NMS). The pathomechanisms and therapeutic modulation of these NMS so far have been understudied. So-called 'premotor symptoms', such as olfactory symptoms, REM sleep disorder, constipation, cardiac dysregulation, and depressive mood can often precede motor symptoms by many years [10]. As PD progresses, in many patients the clinical picture of NMS becomes broader as other neuropsychiatric aspects, urinary symptoms, and dysautonomia can emerge. These symptoms are partly caused by dopaminergic dysfunction and can also be influenced by dopaminergic therapies.
Therefore, not only motor symptoms but also NMS can be treated with dopaminergic drugs. In addition to the l-dopa, which is converted into dopamine in the body, a large number of dopamine agonists are also available as a therapeutic option in PD. These are used preferentially in younger patients and, due to their mechanisms of action on the receptor level and their pharmacokinetics, have a partly different effect profiles in the therapy of the PD.

As PD progresses, the management of motor symptoms becomes increasingly difficult and a monotherapy with oral medication is usually not sufficient to control motor symptoms. After an initial period of uncomplicated PD therapy (so-called 'honeymoon' phase), motor symptoms are alleviated for a shorter time after each medication intake ('wearing off' phenomenon). Furthermore, motor symptoms can occur more rapidly and sometimes unpredictably ('on-off phenomenon'). In the later stages of PD, delays and failures regarding an improvement of motor symptoms after medication can occur [1]. These effects result from a narrower therapeutic window, in which the response to oral dopaminergic medication can correspond to the pharmacokinetics of l-dopa with its plasma median time of 60-90 minutes.

After an exhaustion of conventional oral pharmacotherapy, infusion therapies offer an effective symptomatic therapeutic option. Two infusion therapies are currently available: apomorphine or intrajejunal l-dopa infusion. They enable a more continuous (less pulsatile) application of dopaminergic medication and thus more constant plasma levels of the active substances can be achieved. This requires individually adapted basal flow rates of infusion. Infusion therapies reduce fluctuations and dyskinesia associated with peak concentrations (on-dyskinesia) and the up and down of the concentration of dopaminergic drugs at the striatal receptors (biphasic dyskinesia) [2]. In the advanced stage of PD, both treatment options are superior to oral medication regarding a reduction of motor symptoms as well as an improvement of quality of life [3-8].

In addition to these infusion therapies, the deep brain stimulation (DBS) offers a third therapeutic option for advanced PD. DBS is a surgical, symptomatic therapy option, in which stimulation electrodes are implanted into basal ganglia and motor symptoms are treated with electric current. The aim of this therapy is to modulate pathological neuronal network activity to reduce PD symptoms. This therapy is reversible by switching the stimulation off. The beneficial effects of DBS have been demonstrated in a large number of studies. DBS is superior to medical treatment regarding a reduction of motor symptoms as well as an improvement of the quality of life [9].

In the Department of Neurology of the University Hospital Cologne, these three invasive treatment options are already being applied on a regular basis in patients with advanced stage PD. The number of cases in our department, as one of the national centers for movement disorders and DBS, are about 5-6 patients with apomorphine and 4-5 with l-dopa infusion therapies and 40-50 with deep brain stimulation in PD per year. At present, approximately 50% of these patients undergo studies in which motor symptoms and quality of life are examined but NMS are currently not tested. All other patients are tracked clinically during follow-up visits, but this is not done in standardized study protocols. Against this background, there may therefore be risk of a loss of quality.

A better quality control is therefore necessary: in all patients for NMS and in patients not included in studies so far for quality of life and motor symptoms. Consequently, we are planning a central register for patients with PD treated with conventional pharmacotherapy, apomorphine and l-dopa infusion therapies as well as DBS.

The objective of this registry is quality control by collecting patient data concerning (1) demographics, (2) the use of the therapeutic device system, (3) device-related undesirable side effects, and (4) above-mentioned effects on quality of life, non-motor, and motor symptoms. Furthermore, a quality control is planned for health economic aspects (cost-effectiveness analysis).

This registry can be used for comparisons between treatment options under real conditions of daily life ("real life study"). For the long-term success of therapy, the regular use of the therapy under real operating conditions is the key factor and thus also of scientific interest.

**13. Study aims**

- Quantifying and comparing quality of life, non-motor, and motor symptoms for apomorphine and l-dopa infusion therapies and DBS in advanced PD under real conditions of daily life ("real life study")
- Providing a central registry for patient data for demography, use of the therapeutic device and device-related undesirable side effects
- Characterizing the use of device systems
- Characterizing patient groups which benefit particularly from specific therapeutic device systems regarding quality of life, non-motor, and motor symptoms
- Analyzing health-related factors (incremental cost-effectiveness analysis) for the therapeutic options of the PD

**14. Study plan**

**a. General Information**

The planned study is a prospective registry study for all patients with PD who are treated with apomorphine and l-dopa infusion therapies and DBS.
The choice of the therapeutic procedure in this non-interventional study (NIS) depends on demographic and clinical factors (based on the guidelines of the German Society of Neurology, see table 1) as well as on the preferences of medical personnel, patients, and caregivers [13].

**b. Schedule**

Inclusion of the first patient: 1.3.2011
Inclusion last patient: 1.3.2021
Last patient completing the study: 1.3.2026
End of statistical evaluation: 1.12.2026
Final Report: 01.01.2027

Table 1 Recommendations for the differential indications for apomorphine and l-dopa infusion therapies and deep brain stimulation (adapted according to [13])

| Criterion˙ | Apomorphine infusion | L-dopa infusion | Deep brain stimulation |
|---|---|---|---|
| Age >70 | + | + + | - |
| Mild to moderate dementia | + | + + * | - |
| Severe dementia (MMSE < 10) | + ** | + ** | - - - |
| Tremor (pharmacoresistent) | - | - | + + + |
| Medication induced psychosis | + | + + | + + |
| Testing of procedure | + + + | + | - - - |
| Patient independence | + + | + | + + + |
| Convenience of therapy for patient | - | + | 0 |
| Lack of supporting environment | - - | - - | + |
| Avoiding surgical complications | 0 | - | - - - |

+++ = very suitable, ++ well suited, + = moderately suitable, - = not suitable / unfavorable, - - = very unsuitable / relative contraindication, - - - = absolutely unsuitable / severe contraindication, 0 = not applicable / not specified, * when predisposed to psychoses; ** individual case decision; CAVEAT: agitation!

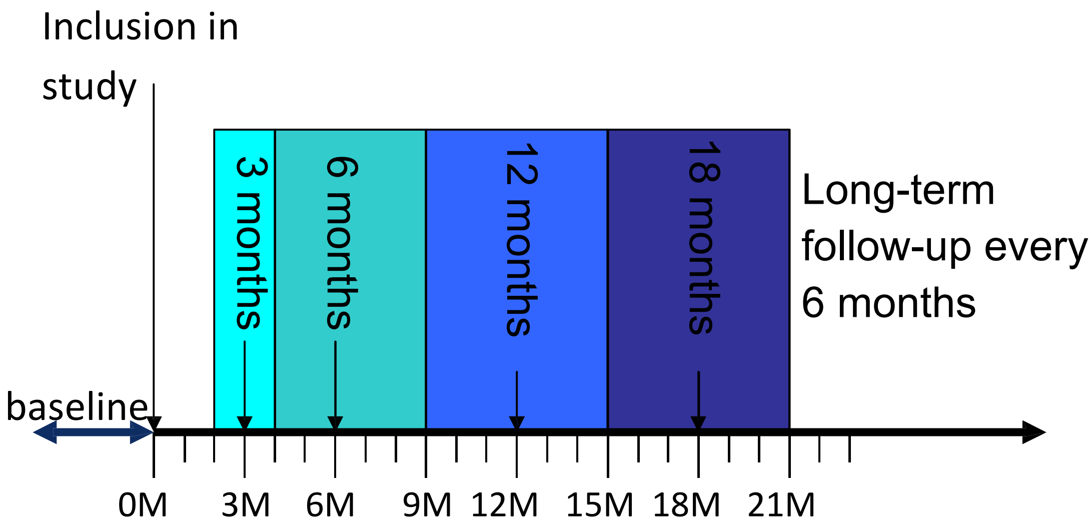

Figure 1: Overview of the timing of the examinations.

**c. Selection of study population / inclusion and exclusion criteria**

Included will be patients with PD treated with a clinical indication for apomorphine and l-dopa infusion therapies and DBS.

All patients will be informed in detail about the background and purpose of the study and will give their informed written consent. On request the discontinuation from the study is possible at any time without giving reasons. The results are subjected to medical confidentiality and are managed and processed after anonymization. Only adults who are able to consent will be examined.

<u>Inclusion criteria of patients with PD:</u>

- Patients of male and female sex with diagnosis of PD according to the British Brain Bank criteria as well as the guidelines of the German Society of Neurology which are, based on the clinical indication, treated with apomorphine infusion, l-dopa infusion or DBS.
- The ability to consent, so that a written consent can be given by the patient.

<u>Exclusion criteria of patients with PD:</u>

- Patients with limited legal capacity, minors, and persons who are placed in an institution on a judicial or administrative order.
- Patients suffering from another serious neurological disease, in addition to the PD.
- Irregularities in MRI, such as ischemia, cerebellar atrophy, etc.
- Previous neurocranial surgery.
- Presence of life-threatening diseases.
- Clinically relevant psychiatric disorders.
- High-degree hearing or visual disturbances which impede the interrogation.

**15. Methodology**

**A) The following scales and questionnaires are collected in standardized assessments:**

1) By medical personnel:

General information: Socio-demographic data, PD-related history, drug therapy, therapy parameters of apomorphine and l-dopa infusion therapy and DBS

Motor symptoms: UPDRS, SCOPA, Hoehn & Yahr stage, Schwab and England Scale
NMS: NMS Scale, CISI-PD, MMST, MoCa, cognitive assessment, verbal fluency test

2) By patient-based self-reported assessment:
Quality of life: PDQ-8
NMS: EQ-5D, HADS, MSS, QUIPS, AES, TAS-20, PDSS-1, patient questionnaire on quality of life and life satisfaction

**B) The above mentioned scales and questionnaires are examined during following visits:**
1) Baseline (during the medical indication assessments, or during the inpatient stay at the start of therapy with apomorphine and l-dopa infusion therapy and DBS)

In further visits as part of the clinical routine at the Department of Neurology:
2) 3 months follow-up (inpatient)
3) 6 months follow-up (outpatient)
4) 12 months follow-up (inpatient)
5) Outpatient follow-ups every 6 months in the “Specialist Clinics for Movement Disorders and DBS” at the Department of Neurology.

**C) Health-economic analyses**
1) Determination of the quality of life years (QALY) using the above-mentioned EQ-5D
2) Cost-effectiveness analysis from a social and cost-carrier perspective

**16. Proposed interventions and burdens on the patient**
General information, quality of life, non-motor, and motor symptoms of patients are collected in the context of the medical indication assessments and on follow-ups. The above mentioned scales and questionnaires are used. No interventions beyond the clinical routine are planned for this study. The patient's burden results from the additional medical history and the assessment of above mentioned scales.

**17. Type of test**
Non-interventional study (NIS): Prospective registry study

**18. Legal requirements and regulations**
General Guideline for the Application of the Drug Testing Guidelines (1995)
Guideline for Good Clinical Practice (1996)
Declaration of Helsinki - Recommendation for physicians involved in biomedical research in humans (Helsinki 1964, Tokyo 1975, Venice 1983, Hong Kong 1989, Somerset West 1996)

**19. Off-label medical products**
Not applicable.

**20. Possible complications and / or risks**

Not applicable.

**21. Risk-benefit assessment**

**Risks:**

The treatment with apomorphine infusion, l-dopa infusion or DBS is based on internal guidelines of the Department of Neurology as per clinical routine. The risks for patients correspond to the general risks of aforementioned therapies. As per clinical routine, neurostimulation and infusion therapies may temporarily and reversibly be paused to check the therapy parameters and functionality of the device systems.

**Benefit-risk assessment:**

A more comprehensive assessment of (1) quality of life, non-motor, and motor symptoms and (2) long-term patient safety in real-life operating conditions is only possible in this "real-life" study design.

The differential indication is currently based on evidence level 4 (expert opinion, [13]). More detailed studies are still pending as to the question which patient groups particularly benefit from which of the three treatment options. The data on quality of life, non-motor, and motor symptoms collected in this registry study can provide information on the differential indication.

**22. Interim evaluation and discontinuation criteria**

Since the present study is not an experimental study but a non-interventional study, an ongoing evaluation of the data is carried out.

Exclusion from the study results:

- In case of an undesirable event, determined by the investigator, which requires treatment discontinuation from apomorphine infusion, l-dopa infusion or DBS
- At the personal request of the patient

**23. Patient information and informed consent form**

See Attachment.

**24. Medical confidentiality**

All those who are dealing with patients within the scope of the study are obliged to maintain medical confidentiality. Compliance with the Federal Data Protection Act is fully guaranteed.

**25. Insurance cover**

Since this is a prospective registry for a non-interventional study with data surveys exclusively in the context of clinically indicated visits, this point is not applicable.

**26. Cost of the study**

The costs are covered by grants of the Movement Disorders and DBS group of the Department of Neurology.

**27. Cooperation with other hospitals / institutes:**

This study will be carried out in collaboration with the Department for Stereotaxy and Functional Neurosurgery. The liaison is Prof. Dr. Veerle Visser-Vandewalle, director of department, who participates in the recruitment of patients with PD.

**28. Signatures**

Dr. med. Haidar Salimi Dafsari

# Patient information in preparation of the verbal information by the doctor for patients with Idiopathic Parkinson's disease

**Registry for the evaluation of treatment of idiopathic Parkinson's disease with the apomorphine pump / duodopa pump / deep brain stimulation**

(Study title: "REAL-DBS-PD: Registry for Apomorphine and L-dopa Infusion Therapies and Deep Brain Stimulation in Patients with Parkinson's Disease")

Dear patient,

We would like to ask for your consent to participate in a registry study. The following pages should inform about the planned registry study and help you decide whether you would like to participate. Please read this patient information carefully. In the subsequent conversation with your doctor you will have the possibility to ask questions.

Your doctor has explained to you that you suffer from idiopathic Parkinson's disease which due to motor fluctuations or dyskinesia, cannot be sufficiently treated exclusively with oral medication, i.e. tablets or pills. As alternatives therapies Apomorphine-and Duodopa pump as well as deep brain stimulation are available. The advantage of these therapy options as compared to tablets and pills is that they can provide a more continuous control of PD symptoms throughout the day and thereby reduce the duration and frequency of motor fluctuations and dyskinesia. The treatment of idiopathic Parkinson's disease with one of these options was assessed and recommended to you based on medical criteria and consultation with you.

The aim of this therapy is to improve quality of life and motor symptoms, i.e. bradykinesia (slowness and small amplitude of movements), rigidity (increased muscle tone) and the tremor (shaking of hands, legs and possibly jaw). Also, so-called 'non-motor' symptoms, i.e. those symptoms of the disease which do not affect the movement system, may possibly be favorably influenced by the therapy. These non-motor symptoms include, e.g., sleep disturbances, heart function, urinary and bowel dysfunctions of, depressive moods and pain.

Our clinic has a profound, long-term experience with these three treatment options. Accurate control of your motor and non-motor symptoms, and quality of life allows us to develop a plan to help you get the best possible settings and usage of your treatment device. The surveys included in this study are standard practice performed in all patients as per clinical routine. We would like to use the results of these examinations for quality control at our Center for Movement Disorders and Deep Brain Stimulation in order to ensure a further improvement of our quality standards.

Purpose of this registry:

The purpose of this registry is quality control by assessments of patient data regarding (1) demographics, (2) use of the therapeutic device system, (3) specific device-related undesirable side effects, and 4) above mentioned effects on quality of life, non-motor, and motor symptoms.

Surveys for the registry:

In this registry we survey scales and questionnaires which take up approximately 45 minutes of your time. No additional investigations are carried out for this study. By participating in the registry, however, you allow us to use the data which is collected for our clinical routine also for scientific purposes.

The participation in the research project is completely voluntary. It can be withdrawn any time and without giving information on reasons for withdrawal of consent. The withdrawal of consent will not result in any disadvantages in your medical treatment or patient care. Should a new neurological or other severe disease develop, a pregnancy occur or the legal capacity become limited, the participation in this study would not possible any more. As a study participant you would be immediately informed if a change of the study design is decided. Should any dangers or increased risks for study participants arise, an early termination of the study is possible.

This research project was approved by the ethical committee of the medical faculty of the University of Cologne. In this investigation a total of 500-600 participants will be enrolled.

**DATA PROTECTION:**

1. All collected data are kept secured according to data protection. This extends beyond a publication of the study results.

2. Name and address for the person responsible for data processing (study leader): Professor Dr. Lars Timmermann, Department of Neurology, University Hospital Cologne, Kerpenerstr. 62, 50937 Cologne, Germany, Tel.: 0221/478-4015

3. Categories of processed data: The following data will be secured for scientific purposes: Imaging data of the brain, data on medical history, clinical data of the neurological examination and psychophysical data.

4. In the pseudonymization of data your name will be replaced by a combination of multiple characters and digits, which constitute a "code", to prevent an identification (see paragraph 3 "Bundesdatenschutzgesetz", Federal Data Protection Bill). However, a pseudonymization will – with the help of a key – allow an attribution of data to a person.

5. The responsible authorities as well as the ethics committee can be granted direct access to the original medical recordings of study participants to the extent of valid legal regulations to oversee clinical testing methods and / or data without violating confidentiality of data of study participants.

Stored data will be deleted after 10 years.

There is a right to receive information on and correct wrongly processed data.

**CONTACT:**

Principal investigators:

Professor Dr. Lars Timmermann

Department of Neurology, University Hospital Cologne, Kerpenerstr. 62, 50937 Cologne

Tel.: +49(0)221 478-4015

Dr. Haidar Salimi Dafsari

Department of Neurology, University Hospital Cologne, Kerpenerstr. 62, 50937 Cologne

Tel.: +49(0)221 478-4015

We would be glad if you take part in the investigation and support our research. If you approve to participate, please fill in and sign the patient approval form in all required fields.

Should you have further questions, please ask your doctor. With pleasure he / she will explain as thoroughly as possible.

Professor Dr. Lars Timmermann Dr. Haidar Salimi Dafsari

I feel sufficiently informed about the research project or the study. My questions were answered and I had sufficient time to consider my decision.

| | |
|---|---|
| ............................................................ | ............................................................................ |
| Place and date, time | Signature of patient |

The informed consent was and all questions were answered.

| | |
|---|---|
| ............................................................ | ............................................................................ |
| Place and date, time | Signature of doctor<br>Study nurse/assistant |

# Registry for Apomorphine and L-Dopa Infusion Therapy and Deep Brain Stimulation in Patients with Parkinson's Disease (REAL-DBS-PD)

## Case Report Form

### Patient-based data

☐Baseline

☐3 Months ☐6 Months ☐12 Months ☐18 Months

☐24 Months ☐30 Months ☐36 Months ☐42 Months

☐48 Months ☐54 Months ☐60 Months

Pat. ID:___________________________

Date:_____________________________

Investigator:______________________

Intervention:______________________

Date of the Intervention:___________

RESULTS

PDQ-8:
EQ-5D:
EQ-5D VAS:
Transition question:
QoL Questionnaire:

NMSQ:
PDSS-1:
HADS-A:
HADS-D:
QUIPS-RS:

TAS-20:
AES:
SRMI:

## PDQ-8

**Due to having Parkinson’s disease, how often during the last month have you…**
**(Please tick one box for each question)**

| | Never | Occasionally | Sometimes | Often | Always |
|---|---|---|---|---|---|
| Had difficulty getting around in public? | ☐ | ☐ | ☐ | ☐ | ☐ |
| Had difficulty dressing yourself? | ☐ | ☐ | ☐ | ☐ | ☐ |
| Felt depressed? | ☐ | ☐ | ☐ | ☐ | ☐ |
| Had problems with close personal relationships? | ☐ | ☐ | ☐ | ☐ | ☐ |
| Had problems with your concentration, for example, when reading or watching TV? | ☐ | ☐ | ☐ | ☐ | ☐ |
| Felt unable to communicate with people properly? | ☐ | ☐ | ☐ | ☐ | ☐ |
| Had painful muscle cramps or spasms? | ☐ | ☐ | ☐ | ☐ | ☐ |
| Felt embarrassed in public due to having Parkinson's disease? | ☐ | ☐ | ☐ | ☐ | ☐ |

## EQ-5D

By placing a tick in one box in each group below, please indicate which statements best describe your own health state today.

**Mobility**

☐ I have no problems in walking

☐ I have some problems in walking about

☐ I am confined to bed

**Self-care**

☐ I have no problems with self-care

☐ I have some problems with washing or dressing myself

☐ I am unable to wash or dress myself

**Usual activities (e.g. work, study, housework or leisure)**

☐ I have no problems with performing my usual activities

☐ I have some problems with performing my usual activities

☐ I am unable to perform my usual activities

**Pain/Discomfort**

☐ I have no pain or discomfort

☐ I have moderate pain or discomfort

☐ I have extreme pain or discomfort

**Anxiety/Depression**

☐ I am not anxious or depressed

☐ I am moderately anxious or depressed

☐ I am extremely anxious or depressed

# Health state today

To help people say how good or bad a health state is, we have drawn a scale (rather like thermometer) on which the best state you can imagine is marked 100 and the worst state you can imagine is marked 0.

We would like you to indicate on the scale how good or bad your own health is today, in your opinion.
Please do this by drawing a line from the box below to whichever point on the scale indicates how good or bad your health state is today.

Your own health state today

Best imaginable health state

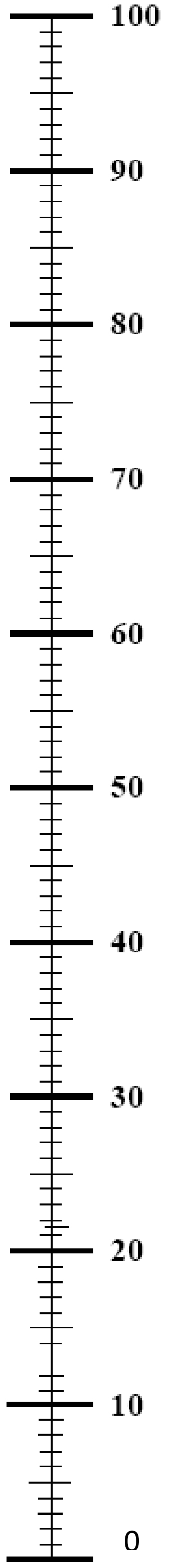


Worst imaginable health state

**Transition Question**

How do you think your Parkinson's disease has changed (improved or worsened) in the past year?

Please, mark "X" in the box of the response that adjust better to you

- ☐ Very much improved
- ☐ Much improved
- ☐ Minimally improved
- ☐ No change
- ☐ Minimally worse
- ☐ Much worse
- ☐ Very much worse

## Questionnaire about QoL and satisfaction

Please mark with a "X" the number representing the most appropriate response to the question

All things considered, how satisfied are you with your **life as a whole?**

**Not at all satisfied** 1 2 3 4 5 6 7 8 9 10 **Very satisfied**

All things considered, how satisfied are you with your **physical health?**

**Not at all satisfied** 1 2 3 4 5 6 7 8 9 10 **Very satisfied**

All things considered, how satisfied are you with your **psychological well-being?**

**Not at all satisfied** 1 2 3 4 5 6 7 8 9 10 **Very satisfied**

All things considered, how satisfied are you with your **social relations?**

**Not at all satisfied** 1 2 3 4 5 6 7 8 9 10 **Very satisfied**

All things considered, how satisfied are you with your **leisure?**

**Not at all satisfied** 1 2 3 4 5 6 7 8 9 10 **Very satisfied**

All things considered, how satisfied are you with the **intervention for Parkinson's disease that you underwent?**

**Not at all satisfied** 1 2 3 4 5 6 7 8 9 10 **Very satisfied**

All things considered, has the Intervention covered your **expectations about?**
**Not at all** 1 2 3 4 5 6 7 8 9 10 **Absolutely**

**NMSQ**

| Have you experienced any of the following in the last month? | Yes | No |
|---|---|---|
| 1. Dribbling of saliva during the daytime | ☐ | ☐ |
| 2. Loss or change in your ability to taste or smell | ☐ | ☐ |
| 3. Difficulty swallowing food or drink or problems with choking | ☐ | ☐ |
| 4. Vomiting or feelings of sickness (nausea) | ☐ | ☐ |
| 5. Constipation (less than 3 bowel movements a week) or having a strain to pass a stool (faeces) | ☐ | ☐ |
| 6. Bowel (fecal) incontinence | ☐ | ☐ |
| 7. Feeling that your bowel emptying is incomplete after having been to the toilet | ☐ | ☐ |
| 8. A sense of urgency to pass urine makes you rush to the toilet | ☐ | ☐ |
| 9. Getting up regularly at night to pass urine | ☐ | ☐ |
| 10. Unexplained pains (not due to known conditions such as arthritis) | ☐ | ☐ |
| 11. Unexplained change in weight (not due to change in diet) | ☐ | ☐ |
| 12. Problems remembering things that have happened recently or forgetting to do things | ☐ | ☐ |
| 13. Loss of interests in what is happening around you or doing things | ☐ | ☐ |
| 14. Seeing or hearing things that you know or are told are not there | ☐ | ☐ |

| Have you experienced any of the following in the last month? | **Yes** | **No** |
|---|---|---|
| 15. Difficulty concentrating or staying focused | ☐ | ☐ |
| 16. Feeling sad, ‚low’ or ‚blue’ | ☐ | ☐ |
| 17. Feeling anxious, frightened or panicky | ☐ | ☐ |
| 18. Feeling less interested in sex or more interested in sex | ☐ | ☐ |
| 19. Finding it more difficult to have sex when you try | ☐ | ☐ |
| 20. Feeling light headed, dizzy or weak standing from sitting or lying | ☐ | ☐ |
| 21. Falling | ☐ | ☐ |
| 22. Finding it difficult to stay awake during activities such as working, driving or eating | ☐ | ☐ |
| 23. Difficulty getting to sleep at night or staying asleep at night | ☐ | ☐ |
| 24. Intense, vivid dreams or frightening dreams | ☐ | ☐ |
| 25. Talking or moving about in your sleep as if you are ‘acting’ out a dream | ☐ | ☐ |
| 26. Unpleasant sensations in your legs at night or while resting, and a feeling that you need to move | ☐ | ☐ |
| 27. Swelling of your legs | ☐ | ☐ |
| 28. Excessive sweating | ☐ | ☐ |
| 29. Double vision | ☐ | ☐ |
| 30. Believing things are happening to you that other people say are not true | ☐ | ☐ |

## PDSS-1

How would you rate the following, based on your experience during the past one week?
Place a cross at the appropriate point on the line.

**1. The overall quality of your night's sleep is?**

awful ●————————● excellent

**2. Do you have difficulty falling asleep each night?**

always ●————————● never

**3. Do you have difficulty staying asleep?**

always ●————————● never

**4. Do you have restlessness of legs or arms at night or in the evening causing disruption of sleep?**

always ●————————● never

**5. Do you fidget in bed?**

always ●————————● never

**6. Do you suffer from distressing dreams at night?**

always ●————————● never

**7. Do you suffer from distressing hallucinations at night (seeing or hearing things that you are told do not exist)?**

always ●————————● never

**8. Do you get up at night to pass urine?**

always ●————————● never

**9. Do you have incontinence of urine because you are unable to move due to "off" symptoms?**

always ●——————————● never

**10. Do you experience numbness or tingling of your arms or legs which wake you from sleep at night?**

always ●——————————● never

**11. Do you have painful muscle cramps in your arms or legs whilst sleeping at night?**

always ●——————————● never

**12. Do you wake early in the morning with painful posturing of arms or legs?**

always ●——————————● never

**13. On waking do you experience tremor?**

always ●——————————● never

**14. Do you feel tired and sleepy after waking in the morning?**

always ●——————————● never

**15. Have you unexpectedly fallen asleep during the day?**

frequently ●——————————● never

## HADS

Please, choose one response from the four given for each interview. The response should be immediate, no thinking too long about the answer. Mark the answer currently describing better your feelings.

---

**I feel tense or 'wound up'**

- ◻ Most of the time
- ◻ A lot of the time
- ◻ From time to time, occasionally
- ◻ Not at all

**I still enjoy the things I used to enjoy**

- ◻ Definitely as much
- ◻ Not quite so much
- ◻ Only a little
- ◻ Hardly at all

**I get a sort of frightened feeling as if something awful is about to happen**

- ◻ Very definitely and quiet badly
- ◻ Yes, but not too badly
- ◻ A little, but it does not worry me
- ◻ Not at all

**I can laugh and see the funny side of things**

- ◻ As much as I always could
- ◻ Not quite so much now
- ◻ Definitely not so much now
- ◻ Not at all

**Worrying thoughts go through my mind**

- ◻ A great deal of the time
- ◻ A lot of the time
- ◻ From time to time, but not too often
- ◻ Only occasionally

**I feel cheerful**

- ◻ Not at all
- ◻ Not often
- ◻ Sometimes
- ◻ Most of the time

**I can sit at ease and feel relaxed**

- ◻ Definitely
- ◻ Usually
- ◻ Not often
- ◻ Not at all

**I feel as if I am slowed down**

- ☐ Nearly all the time
- ☐ Very often
- ☐ Sometimes
- ☐ Not at all

**I get a sort of frightened feeling like 'butterflies' in the stomach:**

- ☐ Not at all
- ☐ Occasionally
- ☐ Quite often
- ☐ Very often

**I have lost interest in my appearance:**

- ☐ Definitely
- ☐ I don’t take as much care as I should
- ☐ I may not take quite as much care
- ☐ I take just as much care as ever

**I feel restless as I have to be on the move:**

- ☐ Very much indeed
- ☐ Quite a lot
- ☐ Not very much
- ☐ Not at all

**I look forward with enjoyment to things:**

□ As much as I ever did

□ Rather less than I used to

□ Definitely less than I used to

□ Hardly at all

**I get sudden feelings of panic**

□ Very often indeed

□ Quite often

□ Not very often

□ Not at all

**I can enjoy a good book or radio or TV program**:

□ Often

□ Sometimes

□ Not very often

□ Very seldom

**QUIPS-RS**

## Instruction Sheet

**TIME FRAME**
**Past 4 weeks**

**DESCRIPTION OF BEHAVIORS**

**A. Gambling** (casinos, internet gambling, lotteries, scratch tickets, betting, or slot or poker machines)

**B. Sex** (making sexual demands on others, promiscuity, prostitution, change in sexual orientation, masturbation, internet or telephone sexual activities, or pornography)

**C. Buying** (too much of the same thing or things that you don't need or use)

**D. Eating** (eating larger amounts or different types of food than in the past, more rapidly than normal, until feeling uncomfortably full, or when not hungry)

**E. Hobbyism** (specific tasks, hobbies or other organized activities, such as writing, painting, gardening, repairing or dismantling things, collecting, computer use, working on projects, etc.)

**F. Punding** (repeating certain simple motor activities, such as cleaning, tidying, handling, examining, sorting, ordering, collecting, hoarding, or arranging objects, etc.)

**G. Medication Use** (consistently taking too much of your Parkinson's medications, or increasing on your own, without medical advice, your overall intake of Parkinson's medications)

**FREQUENCY OF BEHAVIORS**

| | |
|---|---|
| **Never** | **(0) = not at all** |
| **Rarely** | **(1) = infrequently or 1 day/week** |
| **Sometimes** | **(2) = at times or 2-3 days/week** |
| **Often** | **(3) = most of the time or 4-5 days/week** |
| **Very often** | **(4) = nearly always or 6-7 days/week** |

**Please mark the most appropriate answer with an 'X' in each row. Please mark only one answer for each row.**

## QUIPS-RS

Reported by: Patient ☐ Informant ☐ Patient and Informant ☐

**1. How much do you think about the following behaviors (such as having trouble keeping thoughts out of your mind or feeling guilty)?**

| | | | | | |
|---|---|---|---|---|---|
| A. Gambling? | ☐ Never (0) | ☐ Rarely (1) | ☐ Sometimes (2) | ☐ Often (3) | ☐ Very often (4) |
| B. Sex? | ☐ Never (0) | ☐ Rarely (1) | ☐ Sometimes (2) | ☐ Often (3) | ☐ Very often (4) |
| C. Buying? | ☐ Never (0) | ☐ Rarely (1) | ☐ Sometimes (2) | ☐ Often (3) | ☐ Very often (4) |
| D. Eating? | ☐ Never (0) | ☐ Rarely (1) | ☐ Sometimes (2) | ☐ Often (3) | ☐ Very often (4) |
| E. Performing tasks or hobbies? | ☐ Never (0) | ☐ Rarely (1) | ☐ Sometimes (2) | ☐ Often (3) | ☐ Very often (4) |
| F. Repeating simple activities? | ☐ Never (0) | ☐ Rarely (1) | ☐ Sometimes (2) | ☐ Often (3) | ☐ Very often (4) |
| G. Taking your PD medications? | ☐ Never (0) | ☐ Rarely (1) | ☐ Sometimes (2) | ☐ Often (3) | ☐ Very often (4) |

**2. Do you have urges or desires for the following behaviors that you feel are excessive or cause you distress (including becoming restless or irritable when unable participate in them?**

| | | | | | |
|---|---|---|---|---|---|
| A. Gambling? | ☐ Never (0) | ☐ Rarely (1) | ☐ Sometimes (2) | ☐ Often (3) | ☐ Very often (4) |
| B. Sex? | ☐ Never (0) | ☐ Rarely (1) | ☐ Sometimes (2) | ☐ Often (3) | ☐ Very often (4) |
| C. Buying? | ☐ Never (0) | ☐ Rarely (1) | ☐ Sometimes (2) | ☐ Often (3) | ☐ Very often (4) |
| D. Eating? | ☐ Never (0) | ☐ Rarely (1) | ☐ Sometimes (2) | ☐ Often (3) | ☐ Very often (4) |
| E. Performing tasks or hobbies? | ☐ Never (0) | ☐ Rarely (1) | ☐ Sometimes (2) | ☐ Often (3) | ☐ Very often (4) |
| F. Repeating simple activities? | ☐ Never (0) | ☐ Rarely (1) | ☐ Sometimes (2) | ☐ Often (3) | ☐ Very often (4) |
| G. Taking your PD medications? | ☐ Never (0) | ☐ Rarely (1) | ☐ Sometimes (2) | ☐ Often (3) | ☐ Very often (4) |

**3. Do you have difficulty controlling the following behaviors (such as increasing them over time, or having trouble cutting down or stopping them)?**

| | | | | | |
|---|---|---|---|---|---|
| A. Gambling? | ☐ Never (0) | ☐ Rarely (1) | ☐ Sometimes (2) | ☐ Often (3) | ☐ Very often (4) |
| B. Sex? | ☐ Never (0) | ☐ Rarely (1) | ☐ Sometimes (2) | ☐ Often (3) | ☐ Very often (4) |
| C. Buying? | ☐ Never (0) | ☐ Rarely (1) | ☐ Sometimes (2) | ☐ Often (3) | ☐ Very often (4) |
| D. Eating? | ☐ Never (0) | ☐ Rarely (1) | ☐ Sometimes (2) | ☐ Often (3) | ☐ Very often (4) |
| E. Performing tasks or hobbies? | ☐ Never (0) | ☐ Rarely (1) | ☐ Sometimes (2) | ☐ Often (3) | ☐ Very often (4) |
| F. Repeating simple activities? | ☐ Never (0) | ☐ Rarely (1) | ☐ Sometimes (2) | ☐ Often (3) | ☐ Very often (4) |
| G. Taking your PD medications? | ☐ Never (0) | ☐ Rarely (1) | ☐ Sometimes (2) | ☐ Often (3) | ☐ Very often (4) |

**4. Do you engage in activities specifically to continue the following behaviors (such as hiding what you are doing, lying, hoarding things, borrowing from others, accumulating debt, stealing, or being involved in illegal acts?**

| | | | | | |
|---|---|---|---|---|---|
| A. Gambling? | ☐ Never (0) | ☐ Rarely (1) | ☐ Sometimes (2) | ☐ Often (3) | ☐ Very often (4) |
| B. Sex? | ☐ Never (0) | ☐ Rarely (1) | ☐ Sometimes (2) | ☐ Often (3) | ☐ Very often (4) |
| C. Buying? | ☐ Never (0) | ☐ Rarely (1) | ☐ Sometimes (2) | ☐ Often (3) | ☐ Very often (4) |
| D. Eating? | ☐ Never (0) | ☐ Rarely (1) | ☐ Sometimes (2) | ☐ Often (3) | ☐ Very often (4) |
| E. Performing tasks or hobbies? | ☐ Never (0) | ☐ Rarely (1) | ☐ Sometimes (2) | ☐ Often (3) | ☐ Very often (4) |
| F. Repeating simple activities? | ☐ Never (0) | ☐ Rarely (1) | ☐ Sometimes (2) | ☐ Often (3) | ☐ Very often (4) |
| G. Taking your PD medications? | ☐ Never (0) | ☐ Rarely (1) | ☐ Sometimes (2) | ☐ Often (3) | ☐ Very often (4) |

## TAS-20

Using the scale provided as a guide, indicate how much you agree or disagree with each of the following statements by marking respective box with an ‘X’. Give only one answer for each statement.

| | | Strongly disagree | Moderately disagree | Neither disagree nor agree | Moderately agree | Strongly agree |
|---|---|---|---|---|---|---|
| 1. | I am often confused about what emotion I am feeling. | ☐ | ☐ | ☐ | ☐ | ☐ |
| 2. | It is difficult for me to find the right word for my feelings. | ☐ | ☐ | ☐ | ☐ | ☐ |
| 3. | I have physical sensations that even doctors don’t understand. | ☐ | ☐ | ☐ | ☐ | ☐ |
| 4. | I am able to describe my feelings easily. | ☐ | ☐ | ☐ | ☐ | ☐ |
| 5. | I prefer to analyze problems rather than just describe them. | ☐ | ☐ | ☐ | ☐ | ☐ |
| 6. | When I am upset I don’t know if I am sad, frightened or angry. | ☐ | ☐ | ☐ | ☐ | ☐ |
| 7. | I am often puzzled by sensations in my body. | ☐ | ☐ | ☐ | ☐ | ☐ |
| 8. | I prefer to just let things happen rather than to understand why they turned out that way. | ☐ | ☐ | ☐ | ☐ | ☐ |
| 9. | I have feelings that I can’t quite identify. | ☐ | ☐ | ☐ | ☐ | ☐ |
| 10. | Being in touch with emotions is essential. | ☐ | ☐ | ☐ | ☐ | ☐ |

| | | Strongly disagree | Moderately disagree | Neither disagree nor agree | Moderately agree | Strongly agree |
|---|---|---|---|---|---|---|
| 11. | I find it hard to describe how I feel about people. | ☐ | ☐ | ☐ | ☐ | ☐ |
| 12. | People tell me to describe my feelings more. | ☐ | ☐ | ☐ | ☐ | ☐ |
| 13. | I don’t know what’s going inside me. | ☐ | ☐ | ☐ | ☐ | ☐ |
| 14. | I often don’t know why I am angry. | ☐ | ☐ | ☐ | ☐ | ☐ |
| 15. | I prefer talking to people about their activities rather than their feelings. | ☐ | ☐ | ☐ | ☐ | ☐ |
| 16. | I prefer to watch “light” entertainment shows rather than psychological dramas. | ☐ | ☐ | ☐ | ☐ | ☐ |
| 17. | It is difficult for me to reveal my innermost feelings, even to close friends. | ☐ | ☐ | ☐ | ☐ | ☐ |
| 18. | I can feel close to close to someone, even in moments of silence. | ☐ | ☐ | ☐ | ☐ | ☐ |
| 19. | I find examination of my feelings useful in solving personal problems. | ☐ | ☐ | ☐ | ☐ | ☐ |
| 20. | Looking for hidden meanings in movies or plays distracts from their enjoyment. | ☐ | ☐ | ☐ | ☐ | ☐ |

## AES

For each statement mark the box that describes your thoughts, feelings, and activity in the past 4 weeks with an ‘X’.

| | Not at all | Slightly | Somewhat | A lot |
|---|---|---|---|---|
| 1. I am interested in things. | ☐ | ☐ | ☐ | ☐ |
| 2. I get things done during the day. | ☐ | ☐ | ☐ | ☐ |
| 3. Getting things started on my own is important to me. | ☐ | ☐ | ☐ | ☐ |
| 4. I am interested in having new experiences. | ☐ | ☐ | ☐ | ☐ |
| 5. I am interested in learning new things. | ☐ | ☐ | ☐ | ☐ |
| 6. I put little effort into anything. | ☐ | ☐ | ☐ | ☐ |
| 7. I approach life with intensity. | ☐ | ☐ | ☐ | ☐ |
| 8. Seeing a job through to the end is important to me. | ☐ | ☐ | ☐ | ☐ |
| 9. I spend time doing things that interest me. | ☐ | ☐ | ☐ | ☐ |
| 10. Someone has to tell me what to do each day. | ☐ | ☐ | ☐ | ☐ |
| 11. I am less concerned about my problems than I should be. | ☐ | ☐ | ☐ | ☐ |
| 12. I have friends. | ☐ | ☐ | ☐ | ☐ |
| 13. Getting together with friends is important to me. | ☐ | ☐ | ☐ | ☐ |
| 14. When something good happens, I get excited. | ☐ | ☐ | ☐ | ☐ |
| 15. I have an accurate understanding of my problems. | ☐ | ☐ | ☐ | ☐ |
| 16. Getting things done during the day is important to me. | ☐ | ☐ | ☐ | ☐ |
| 17. I have initiative. | ☐ | ☐ | ☐ | ☐ |
| 18. I have motivation. | ☐ | ☐ | ☐ | ☐ |

## SRMI

Have you experienced the statements below in the past 4 weeks? Please relate to your “normal” behavior and answer “Yes” or “No” by placing a tick in one box.

| | Question | Yes | No |
|---|---|---|---|
| (1) | I had more energy. | ☐ | ☐ |
| (2) | I had trouble sitting still. | ☐ | ☐ |
| (3) | I drove faster. | ☐ | ☐ |
| (4) | I drank more alcoholic beverages. | ☐ | ☐ |
| (5) | I changed clothes several times a day. | ☐ | ☐ |
| (6) | I wore brighter clothes/ make-up. | ☐ | ☐ |
| (7) | I played music louder. | ☐ | ☐ |
| (8) | I ate faster than usual. | ☐ | ☐ |
| (9) | I ate more than usual. | ☐ | ☐ |
| (10) | I slept fewer hours than usual. | ☐ | ☐ |
| (11) | I started things that I didn’t finish. | ☐ | ☐ |
| (12) | I gave away my own possessions. | ☐ | ☐ |
| (13) | I bought gifts for people. | ☐ | ☐ |
| (14) | I spent money more freely. | ☐ | ☐ |
| (15) | I accumulated debts. | ☐ | ☐ |
| (16) | I made unwise business decisions. | ☐ | ☐ |
| (17) | I partied more. | ☐ | ☐ |
| (18) | I enjoyed flirting. | ☐ | ☐ |
| (19) | I masturbated more often. | ☐ | ☐ |
| (20) | I was more interested in sex than usual. | ☐ | ☐ |
| (21) | I had sex with people that I usually wouldn’t have sex with. | ☐ | ☐ |
| (22) | I spent more time on the phone. | ☐ | ☐ |
| (23) | I spoke louder than usual. | ☐ | ☐ |

| | Question | Yes | No |
|---|---|---|---|
| (24) | I spoke so fast that people said they couldn’t understand me. | ☐ | ☐ |
| (25) | I enjoyed punning or rhyming. | ☐ | ☐ |
| (26) | I butted into conversations. | ☐ | ☐ |
| (27) | I spoke on and on and couldn’t be interrupted. | ☐ | ☐ |
| (28) | I enjoyed being the center of attention. | ☐ | ☐ |
| (29) | I liked to joke and laugh. | ☐ | ☐ |
| (30) | People found me entertaining. | ☐ | ☐ |
| (31) | I felt as if I was on top of the world. | ☐ | ☐ |
| (32) | I was more cheerful than my usual self. | ☐ | ☐ |
| (33) | Other people got on my nerves. | ☐ | ☐ |
| (34) | I was getting into arguments. | ☐ | ☐ |
| (35) | I had so many ideas that I couldn’t get around to doing them all. | ☐ | ☐ |
| (36) | My thoughts raced through my mind. | ☐ | ☐ |
| (37) | I couldn’t concentrate on a single topic for longer than a minute. | ☐ | ☐ |
| (38) | I thought I was an especially important person. | ☐ | ☐ |
| (39) | I thought I could change the world. | ☐ | ☐ |
| (40) | I thought I was right most of the time. | ☐ | ☐ |
| (41) | I thought I was superior to others. | ☐ | ☐ |
| (42) | I wanted to take on jobs that I was not trained to handle. | ☐ | ☐ |
| (43) | I thought I knew what other people were thinking. | ☐ | ☐ |
| (44) | I thought other people knew what I was thinking. | ☐ | ☐ |
| (45) | I thought someone wanted to harm me. | ☐ | ☐ |
| (46) | I heard voices when people weren’t there. | ☐ | ☐ |
| (47) | I had false beliefs concerning who I was. | ☐ | ☐ |
| (48) | I knew I was getting ill. | ☐ | ☐ |

## Motor diary 1 (please fill in!)

Please indicate your motor state for 24 hours by marking respective box with an 'X'. Give only one answer for each time period.

Motor diary for the following date: ____/______/___________

| | ON | ON with troublesome dyskinesia | OFF | Asleep |
|---|---|---|---|---|
| 00:00 – 01:00 | ☐ | ☐ | ☐ | ☐ |
| 01:00 – 02:00 | ☐ | ☐ | ☐ | ☐ |
| 02:00 – 03:00 | ☐ | ☐ | ☐ | ☐ |
| 03:00 – 04:00 | ☐ | ☐ | ☐ | ☐ |
| 04:00 – 05:00 | ☐ | ☐ | ☐ | ☐ |
| 05:00 – 06:00 | ☐ | ☐ | ☐ | ☐ |
| 06:00 – 07:00 | ☐ | ☐ | ☐ | ☐ |
| 07:00 – 08:00 | ☐ | ☐ | ☐ | ☐ |
| 08:00 – 09:00 | ☐ | ☐ | ☐ | ☐ |
| 09:00 – 10:00 | ☐ | ☐ | ☐ | ☐ |
| 10:00 – 11:00 | ☐ | ☐ | ☐ | ☐ |
| 11:00 – 12:00 | ☐ | ☐ | ☐ | ☐ |
| 12:00 – 13:00 | ☐ | ☐ | ☐ | ☐ |
| 13:00 – 14:00 | ☐ | ☐ | ☐ | ☐ |
| 14:00 – 15:00 | ☐ | ☐ | ☐ | ☐ |
| 15:00 – 16:00 | ☐ | ☐ | ☐ | ☐ |
| 16:00 – 17:00 | ☐ | ☐ | ☐ | ☐ |
| 17:00 – 18:00 | ☐ | ☐ | ☐ | ☐ |
| 18:00 – 19:00 | ☐ | ☐ | ☐ | ☐ |
| 19:00 – 20:00 | ☐ | ☐ | ☐ | ☐ |
| 20:00 – 21:00 | ☐ | ☐ | ☐ | ☐ |
| 21:00 – 22:00 | ☐ | ☐ | ☐ | ☐ |
| 22:00 – 23:00 | ☐ | ☐ | ☐ | ☐ |
| 23:00 – 00:00 | ☐ | ☐ | ☐ | ☐ |

## Motor diary 2 (optional)

Please indicate your motor state for 24 hours by marking respective box with an 'X'. Give only one answer for each time period.

Motor diary for the following date: ____/______/___________

| | ON | ON with troublesome dyskinesia | OFF | Asleep |
|---|---|---|---|---|
| 00:00 – 01:00 | ☐ | ☐ | ☐ | ☐ |
| 01:00 – 02:00 | ☐ | ☐ | ☐ | ☐ |
| 02:00 – 03:00 | ☐ | ☐ | ☐ | ☐ |
| 03:00 – 04:00 | ☐ | ☐ | ☐ | ☐ |
| 04:00 – 05:00 | ☐ | ☐ | ☐ | ☐ |
| 05:00 – 06:00 | ☐ | ☐ | ☐ | ☐ |
| 06:00 – 07:00 | ☐ | ☐ | ☐ | ☐ |
| 07:00 – 08:00 | ☐ | ☐ | ☐ | ☐ |
| 08:00 – 09:00 | ☐ | ☐ | ☐ | ☐ |
| 09:00 – 10:00 | ☐ | ☐ | ☐ | ☐ |
| 10:00 – 11:00 | ☐ | ☐ | ☐ | ☐ |
| 11:00 – 12:00 | ☐ | ☐ | ☐ | ☐ |
| 12:00 – 13:00 | ☐ | ☐ | ☐ | ☐ |
| 13:00 – 14:00 | ☐ | ☐ | ☐ | ☐ |
| 14:00 – 15:00 | ☐ | ☐ | ☐ | ☐ |
| 15:00 – 16:00 | ☐ | ☐ | ☐ | ☐ |
| 16:00 – 17:00 | ☐ | ☐ | ☐ | ☐ |
| 17:00 – 18:00 | ☐ | ☐ | ☐ | ☐ |
| 18:00 – 19:00 | ☐ | ☐ | ☐ | ☐ |
| 19:00 – 20:00 | ☐ | ☐ | ☐ | ☐ |
| 20:00 – 21:00 | ☐ | ☐ | ☐ | ☐ |
| 21:00 – 22:00 | ☐ | ☐ | ☐ | ☐ |
| 22:00 – 23:00 | ☐ | ☐ | ☐ | ☐ |
| 23:00 – 00:00 | ☐ | ☐ | ☐ | ☐ |

## Motor diary 3 (optional)

Please indicate your motor state for 24 hours by marking respective box with an 'X'. Give only one answer for each time period.

Motor diary for the following date: ____/______/___________

| | ON | ON with troublesome dyskinesia | OFF | Asleep |
|---|---|---|---|---|
| 00:00 – 01:00 | ☐ | ☐ | ☐ | ☐ |
| 01:00 – 02:00 | ☐ | ☐ | ☐ | ☐ |
| 02:00 – 03:00 | ☐ | ☐ | ☐ | ☐ |
| 03:00 – 04:00 | ☐ | ☐ | ☐ | ☐ |
| 04:00 – 05:00 | ☐ | ☐ | ☐ | ☐ |
| 05:00 – 06:00 | ☐ | ☐ | ☐ | ☐ |
| 06:00 – 07:00 | ☐ | ☐ | ☐ | ☐ |
| 07:00 – 08:00 | ☐ | ☐ | ☐ | ☐ |
| 08:00 – 09:00 | ☐ | ☐ | ☐ | ☐ |
| 09:00 – 10:00 | ☐ | ☐ | ☐ | ☐ |
| 10:00 – 11:00 | ☐ | ☐ | ☐ | ☐ |
| 11:00 – 12:00 | ☐ | ☐ | ☐ | ☐ |
| 12:00 – 13:00 | ☐ | ☐ | ☐ | ☐ |
| 13:00 – 14:00 | ☐ | ☐ | ☐ | ☐ |
| 14:00 – 15:00 | ☐ | ☐ | ☐ | ☐ |
| 15:00 – 16:00 | ☐ | ☐ | ☐ | ☐ |
| 16:00 – 17:00 | ☐ | ☐ | ☐ | ☐ |
| 17:00 – 18:00 | ☐ | ☐ | ☐ | ☐ |
| 18:00 – 19:00 | ☐ | ☐ | ☐ | ☐ |
| 19:00 – 20:00 | ☐ | ☐ | ☐ | ☐ |
| 20:00 – 21:00 | ☐ | ☐ | ☐ | ☐ |
| 21:00 – 22:00 | ☐ | ☐ | ☐ | ☐ |
| 22:00 – 23:00 | ☐ | ☐ | ☐ | ☐ |
| 23:00 – 00:00 | ☐ | ☐ | ☐ | ☐ |

# Registry for Apomorphine and L-Dopa Infusion Therapy and Deep Brain Stimulation in Patients with Parkinson's Disease (REAL-DBS-PD)

## Case Report Form

### Clinician-based data

☐Baseline

| | | | |
|---|---|---|---|
| ☐3 Months | ☐6 Months | ☐12 Months | ☐18 Months |
| ☐24 Months | ☐30 Months | ☐36 Months | ☐42 Months |
| ☐48 Months | ☐54 Months | ☐60 Months | |

Pat. ID:__________________________ Date:___________________________

Investigator:______________________ Intervention:______________________

Date of the Intervention:___________

### RESULTS

UPDRS-I: -II: -III: -IV:
Hoehn & Yahr:
Schwab & England:
SCOPA-A: -B: -C:
CISI-PD:

NMSS:
MMST:
MoCa:
Cognitive assessment:
Word fluency:

## Demography

Date of birth:___________ Age:___________ ☐ ♂ ☐ ♀

Civil Status:
- ☐ Single
- ☐ Married
- ☐ Widow
- ☐ Separated/ divorced

Activity
- ☐ Employee or autonomous
- ☐ Retired/pensioner
- ☐ Housewife
- ☐ Student
- ☐ Unemployed
- ☐ Other:____________________

Educative level:
- ☐ No education at all
- ☐ Elementary school
- ☐ High school
- ☐ University or similar

Years of education:__________

---

## PD-related history

Age at PD diagnosis:________

Age at PD onset:___________

Age at manifestation of motor fluctuations (dyskinesia/ ON-OFF fluctuations):________

---

## Oral medication*

Levodopa:______________________________

Dopamine agonist:______________________________

______________________________

COMT-Inhibitor:______________________________

MAO-B-Inhibitor:______________________________

Amantadine:______________________________

Others (incl. psychotropic medication):______________________________

______________________________

______________________________

* Documentation pattern: L-dopa/Carbidopa 100/25 mg 1-1-1

## Injection/infusion therapy

Type:______________________________

Flow rate:______________________________

Morning dose:______________________________

Bolus (frequency and amount):___________

Infusion duration (h/day):_______________

## Deep brain stimulation

Stimulation parameters

______________________________

______________________________

______________________________

______________________________

Impedances

______________________________

______________________________

# UPDRS

Notes:

## I. Mentation, Behavior and Mood

**1. Intellectual Impairment** ☐

0 = None.
1 = Mild. Consistent forgetfulness with partial recollection of events and no other difficulties.
2 = Moderate memory loss, with disorientation and moderate difficulty handling complex problems. Mild but definite impairment of function at home with need of occasional prompting.
3 = Severe memory loss with disorientation for time and often to place. Severe impairment in handling problems.
4 = Severe memory loss with orientation preserved to person only. Unable to make judgements or solve problems. Requires much help with personal care. Cannot be left alone at all

**2. Thought Disorder** ☐

(Due to dementia or drug intoxication)
0 = None.
1 = Vivid dreaming.
2 = "Benign" hallucinations with insight retained.
3 = Occasional to frequent hallucinations or delusions; without insight; could interfere with daily activities.
4 = Persistent hallucinations, delusions, or florid psychosis. Not able to care for self.

**3. Depression** ☐

0 = None.
1 = Periods of sadness or guilt greater than normal, never sustained for days or weeks.
2 = Sustained depression (week or more).
3 = Sustained depression with vegetative symptoms (insomnia, anorexia, weight loss, loss of interest).
4 = Sustained depression with vegetative symptoms and suicidal thoughts or intent.

**4. Motivation/Initiative** ☐

0 = Normal.
1 = Less assertive than usual; more passive.
2 = Loss of initiative or disinterest in elective (nonroutine) activities.
3 = Loss of initiative or disinterest in day to day (routine) activities.
4 = Withdrawn, complete loss of motivation.

**I. Total** ☐

## II. Activities of Daily Living

ON OFF

**5. Speech**
0 = Normal.
1 = Mildly affected. No difficulty being understood.
2 = Moderately affected. Sometimes asked to repeat statements.
3 = Severely affected. Frequently asked to repeat statements.
4 = Unintelligible most of the time.

**6. Salivation**
0 = Normal.
1 = Slight but definite excess of saliva in mouth; may have night time drooling.
2 = Moderately excessive saliva; may have minimal drooling.
3 = Marked excess of saliva with some drooling.
4 = Marked drooling, requires constant tissue or handkerchief.

**7. Swallowing**
0 = Normal.
1 = Rare choking.
2 = Occasional choking.
3 = Requires soft food.
4 = Requires NG tube or gastrotomy feeding.

**8. Handwriting**
0 = Normal.
1 = Slightly slow or small.
2 = Moderately slow or small; all words are legible.
3 = Severely affected; not all words are legible.
4 = The majority of words are not legible.

**9. Cutting Food and Handling Utensils**
0 = Normal.
1 = Somewhat slow and clumsy, but no help needed.
2 = Can cut most foods, although clumsy and slow; some help needed.
3 = Food must be cut by someone, but can still feed slowly.
4 = Needs to be fed.

**10. Dressing**
0 = Normal.
1 = Somewhat slow, but no help needed.
2 = Occasional assistance with buttoning, getting arms in sleeves.
3 = Considerable help required, but can do some things alone.
4 = Helpless.

**11. Hygiene**
0 = Normal.
1 = Somewhat slow, but no help needed.
2 = Needs help to shower or bathe; or very slow in hygienic care
3 = Requires assistance for washing, brushing teeth, combing hair, going to bathroom.
4 = Foley catheter or other mechanical aids.

ON OFF

**12. Turning in Bed and Adjusting Bed Clothes**
0 = Normal.
1 = Somewhat slow and clumsy, but no help needed.
2 = Can turn alone or adjust sheets, but with great difficulty.
3 = Can initiate, but not turn or adjust sheets alone.
4 = Helpless.

**13. Falling (Unrelated to Freezing)**
0 = None.
1 = Rare falling.
2 = Occasionally falls, less than once per day.
3 = Falls an average of once daily.
4 = Falls more than once daily.

**14. Freezing when Walking**
0 = None.
1 = Rare freezing when walking; may have start hesitation.
2 = Occasional freezing when walking.
3 = Frequent freezing. Occasionally falls from freezing.
4 = Frequent falls from freezing.

**15. Walking**
0 = Normal.
1 = Mild difficulty. May not swing arms or may tend to drag leg.
2 = Moderate difficulty, but requires little or no assistance.
3 = Severe disturbance of walking, requiring assistance.
4 = Cannot walk at all, even with assistance.

**16. Tremor**
(Symptomatic complaint of tremor in any part of body.)
0 = Absent.
1 = Slight and infrequently present.
2 = Moderate; bothersome to patient.
3 = Severe; interferes with many activities.
4 = Marked; interferes with most activities.

**17. Sensory Complaints Related to Parkinsonism**
0 = None.
1 = Occasionally has numbness, tingling, or mild aching.
2 = Frequently has numbness, tingling, or aching; not distressing.
3 = Frequent painful sensations.
4 = Excruciating pain.

**II. Total** **ON** ☐ **OFF** ☐

## III. Motor Examination

ON OFF

**18. Speech**

0 = Normal.
1 = Slight loss of expression, diction and/or volume.
2 = Monotone, slurred but understandable; moderately impaired.
3 = Marked impairment, difficult to understand.
4 = Unintelligible

**19. Facial Expression**

0 = Normal.
1 = Minimal hypomimia, could be normal "Poker Face."
2 = Slight but definitely abnormal diminution of facial expression
3 = Moderate hypomimia; lips parted some of the time.
4 = Masked or fixed facies with severe or complete loss of facial expression; lips parted 1/4inch or more.

**20. Tremor at Rest (head, upper and lower extremities)**

0 = Absent.
1 = Slight and infrequently present.
2 = Mild in amplitude and persistent. Or moderate in amplitude, but only intermittently present.
3 = Moderate in amplitude and present most of the time.
4 = Marked in amplitude and present most of the time.

Face
Right hand
Left hand
Right foot
Left foot

**21. Action or Postural Tremor of Hands**

0 = Absent.
1 = Slight; present with action.
2 = Moderate in amplitude, present with action.
3 = Moderate in amplitude with posture holding as well as action.
4 = Marked in amplitude; interferes with feeding.

Right
Left

**22. Rigidity**

(Judged on passive movement of major joints with patient relaxed in sitting position. Cogwheeling to be ignored.)

0 = Absent.
1 = Slight or detectable only when activated by mirror or other movements.
2 = Mild to moderate.
3 = Marked, but full range of motion easily achieved.
4 = Severe, range of motion achieved with difficulty.

Neck
Right upper extremities
Left upper extremities
Right lower extremities
Left lower extremities

**23. Finger Taps**

(Patient taps thumb with index finger in rapid succession.)

0 = Normal.
1 = Mild slowing and/or reduction in amplitude.
2 = Moderately impaired. Definite and early fatiguing. May have occasional arrests in movement.
3 = Severely impaired. Frequent hesitation in initiating movements or arrests in ongoing movement.
4 = Can barely perform the task.

Right
Left

**24. Hand Movements**

(Patient opens and closes hands in rapid succession.)

0 = Normal.
1 = Mild slowing and/or reduction in amplitude.
2 = Moderately impaired. Definite and early fatiguing. May have occasional arrests in movement.
3 = Severely impaired. Frequent hesitation in initiating movements or arrests in ongoing movement.
4 = Can barely perform the task.

Right
Left

## III. Motor Examination

ON OFF

**25. Rapid Alternating Movements of Hands**

(Pronation-supination movements of hands, vertically and horizontally, with as large an amplitude as possible, both hands simultaneously.)

Right
Left

0 = Normal.
1 = Mild slowing and/or reduction in amplitude.
2 = Moderately impaired. Definite and early fatiguing. May have occasional arrests in movement.
3 = Severely impaired. Frequent hesitation in initiating movements or arrests in ongoing movement.
4 = Can barely perform the task.

**26. Leg Agility**

(Patient taps heel on the ground in rapid succession picking up entire leg. Amplitude should be at least 3 inches.)

Right
Left

0 = Normal.
1 = Mild slowing and/or reduction in amplitude.
2 = Moderately impaired. Definite and early fatiguing. May have occasional arrests in movement.
3 = Severely impaired. Frequent hesitation in initiating movements or arrests in ongoing movement.
4 = Can barely perform the task.

**27. Arising from Chair**

(Patient attempts to rise from a straightbacked chair, with arms folded across chest.)

0 = Normal.
1 = Slow; or may need more than one attempt.
2 = Pushes self up from arms of seat.
3 = Tends to fall back and may have to try more than one time, but can get up without help.
4 = Unable to arise without help.

**28. Posture**

0 = Normal erect.
1 = Not quite erect, slightly stooped posture; could be normal for older person.
2 = Moderately stooped posture, definitely abnormal; can be slightly leaning to one side.
3 = Severely stooped posture with kyphosis; can be moderately leaning to one side.
4 = Marked flexion with extreme abnormality of posture.

**29. Gait**

0 = Normal.
1 = Walks slowly, may shuffle with short steps, but no festination (hastening steps) or propulsion.
2 = Walks with difficulty, but requires little or no assistance; may have some festination, short steps, or propulsion.
3 = Severe disturbance of gait, requiring assistance.
4 = Cannot walk at all, even with assistance.

**30. Postural Stability**

(Response to sudden, strong posterior displacement produced by pull on shoulders while patient erect with eyes open and feet slightly apart. Patient is prepared.)

0 = Normal.
1 = Retropulsion, but recovers unaided.
2 = Absence of postural response; would fall if not caught by examiner.
3 = Very unstable, tends to lose balance spontaneously.
4 = Unable to stand without assistance.

.

## III. Motor Examination

ON OFF

**31. Body Bradykinesia and Hypokinesia**

(Combining slowness, hesitancy, decreased arm swing, small amplitude, and poverty of movement in general.)

0 = None.
1 = Minimal slowness, giving movement a deliberate character; could be normal for some persons. Possibly reduced amplitude.
2 = Mild degree of slowness and poverty of movement which is definitely abnormal. Alternatively, some reduced amplitude.
3 = Moderate slowness, poverty or small amplitude of movement.
4 = Marked slowness, poverty or small amplitude of movement.

**III. Total ON OFF**

## IV. Complications of Therapy

### A. Dyskinesien

**32. Duration: What proportion of the waking day are dyskinesias present?**

(Historical information.)

0 = None.
1 = 1 - 25% of day.
2 = 26 - 50% of day.
3 = 51 - 75% of day.
4 = 76 - 100% of day.

**33. Disability: How disabling are the dyskinesias?**

(Historical information; may be modified by office examination.)

0 = Not disabling.
1 = Mildly disabling.
2 = Moderately disabling.
3 = Severely disabling.
4 = Completely disabled.

**34. Painful Dyskinesias: How painful are the dyskinesias?**

0 = No painful dyskinesias.
1 = Slight.
2 = Moderate.
3 = Severe.
4 = Marked.

**35. Presence of Early Morning Dystonia**

(Historical information.)

0 = No.
1 = Yes.

### B. Clinical Fluctuations

**36. Are "off" periods predictable?**

0 = No.
1 = Yes.

**37. Are "off" periods unpredictable?**

0 = No.
1 = Yes.

**38. Do "off" periods come on suddenly, within a few seconds?**

0 = No.
1 = Yes.

**39 What proportion of the waking day is the patient "off" on average?**

0 = None.
1 = 1 - 25% of day.
2 = 26 - 50% of day.
3 = 51 - 75% of day.
4 = 76 - 100% of day.

### C. Other Complications

**40. Does the patient have anorexia, nausea, or vomiting?**

0 = No.
1 = Yes.

**41. Any sleep disturbances, such as insomnia or hypersomnolence?**

0 = No.
1 = Yes.

**42. Does the patient have symptomatic orthostasis?**

(Record the patient's blood pressure, height and weight on the scoring form)

0 = No.
1 = Yes.

**Scores:**

**Part 1:** _________
**Part 2:** _________
**Part 3:** _________
**Part 4:** _________ **Total:** _____________

# V. Modified Hoehn and Yahr Staging

| | | ON | OFF |
|---|---|---|---|
| Stage 0 | No signs of disease. | | |
| Stage 1 | Unilateral disease. | | |
| Stage 1,5 | Unilateral plus axial involvement | | |
| Stage 2 | Bilateral disease, without impairment of balance. | | |
| Stage 2,5 | Mild bilateral disease, with recovery on pull test | | |
| Stage 3 | Mild to moderate bilateral disease; some postural instability; physically independent. | | |
| Stage 4 | Severe disability; still able to walk or stand unassisted | | |
| Stage 5 | Wheelchair bound or bedridden unless aided. | | |

# VI. Schwab and England Activities of Daily Living Scale

| | | ON | OFF |
|---|---|---|---|
| 100 % | Completely independent. Able to do all chores without slowness, difficulty or impairment. Essentially normal.<br>Unaware of any difficulty. | | |
| 90 % | Completely independent. Able to do all chores with some degree of slowness, difficulty and impairment.<br>Might take twice as long. Beginning to be aware of difficulty | | |
| 80 % | Completely independent in most chores. Takes twice as long. Conscious of difficulty and slowness. | | |
| 70 % | Not completely independent. More difficulty with some chores. Three to four times as long in some.<br>Must spend a large part of the day with chores. | | |
| 60 % | Some dependency. Can do most chores, but exceedingly slowly and with much effort. Errors; some impossible. | | |
| 50 % | More dependent. Help with half, slower, etc. Difficulty with everything. | | |
| 40 % | Very dependent. Can assist with all chores, but few alone. | | |
| 30 % | With effort, now and then does a few chores alone or begins alone. Much help needed. | | |
| 20 % | Nothing alone. Can be a slight help with some chores. Severe invalid. | | |
| 10 % | Totally dependent, helpless. Complete invalid. | | |
| 0 % | Vegetative functions such as swallowing, bladder and bowel functions are not functioning. Bedridden. | | |

# SCOPA-MOTOR SCORE

## A Motor evaluation

### Clinical examination

1. **Rest tremor**
   Assess each arm separately during 20 seconds; hands rest on thighs; if tremor is not evident at rest, try to keep the patient attentive, e.g. by having him/her count backwards with eyes closed.
   0 = absent
   1 = small amplitude (< 1 cm) occurring spontaneously, or obtained only while keeping patient attentive (any amplitude)
   2 = moderate amplitude (1-4 cm), occurring spontaneously
   3 = large amplitude (≥ 4 cm), occurring spontaneously

   ☐ right ☐ left

2. **Postural tremor**
   Check with arms outstretched, pronated and semipronated, and with index fingers of both hands almost touching each other (elbows flexed); assess each position during 20 seconds.
   0 = absent
   1 = small amplitude (< 1 cm)
   2 = moderate amplitude (1-4 cm)
   3 = large amplitude (≥ 4 cm)

   ☐ right ☐ left

3. **Rapid alternating movements of hands**
   Rapid alternating pronation/supination movements of upper hand, each time slapping the palm of the horizontally held lower hand during 20 seconds; each hand separately.
   0 = normal
   1 = slow execution, or mild slowing and/or reduction in amplitude
   2 = definite and early fatiguing; may have occasional arrests
   3 = hesitation in initiating movement or frequent arrests in ongoing movements, or can barely perform task

   ☐ right ☐ left

4. **Rigidity**
   Assess passive movements of elbow and wrist over full range, with the patient relaxed in sitting position; ignore cogwheeling; check each arm separately.
   0 = absent
   1 = mild rigidity over full range, no difficulty reaching end positions
   2 = moderate rigidity, some difficulties reaching end positions
   3 = severe rigidity, considerable difficulties reaching end positions

   ☐ right ☐ left

5. **Rise from chair**
   Patient is instructed to fold arms across chest; use straight back chair.
   - 0 = normal
   - 1 = slowly; does not need arms to get up
   - 2 = needs arms to get up (can get up without help)
   - 3 = unable to rise (without help)

6. **Postural instability**
   Stand behind the patient and pull patient backwards, while s/he is standing erect with eyes open and feet spaced slightly apart; patient is not prepared.
   - 0 = normal, may take up to 2 steps to recover
   - 1 = takes 3 or more steps; recovers unaided
   - 2 = would fall if not caught
   - 3 = spontaneous tendency to fall or unable to stand unaided

7. **Gait**
   Assess gait pattern; use walking aid or offer assistance, if necessary.
   - 0 = normal
   - 1 = mild slowing and/or reduction of step height or length; does not shuffle
   - 2 = severe slowing, or shuffles or has festination
   - 3 = unable to walk.

8. **Speech**
   - 0 = normal
   - 1 = slight loss of expression, diction and/or volume
   - 2 = slurred; not always intelligible
   - 3 = unintelligible always or most of the time

## Historical information

9. **Freezing during 'on'**
   Freezing is characterized by hesitation when trying to start walking or 'gluing' to the ground while walking.
   - 0 = absent
   - 1 = start hesitation only, occasionally present
   - 2 = frequently present, may have freezing when walking
   - 3 = severe freezing when walking

10. **Swallowing**
    - 0 = normal
    - 1 = some difficulty or slow; does not choke; normal diet
    - 2 = sometimes chokes; may require soft food
    - 3 = chokes frequently; may require soft food or alternative method of food intake

## B. Activities of Daily Living

**11. Speech**

0 = normal
1 = some difficulty; may sometimes be asked to repeat sentences
2 = considerable difficulty; frequently asked to repeat sentences
3 = unintelligible most of the time

**12. Feeding (cutting, filling cup, etc.)**

0 = normal
1 = some difficulty or slow; does not need assistance
2 = considerable difficulty; needs some assistance
3 = needs almost complete or complete assistance.

**13. Dressing**

0 = normal
1 = some difficulty or slow; does not need assistance
2 = considerable difficulty; needs some assistance (e.g. with buttoning, getting arms into sleeves)
3 = needs almost complete or complete assistance

**14. Hygiene (washing, combing hair, shaving, brushing teeth, using toilet)**

0 = normal
1 = some difficulty or slow; does not need assistance
2 = considerable difficulty; needs some assistance
3 = needs almost complete or complete assistance

**15. Changing position (turning over in bed, getting up out of bed, getting up out of a chair, turning around when standing)**

0 = normal
1 = some difficulty or slow; does not need assistance with any change of position
2 = considerable difficulty; may need assistance with one or more changes of position
3 = needs almost complete or complete assistance with one or more changes of position

**16. Walking**

0 = normal
1 = some difficulty or slow; does not need assistance or walking aid
2 = considerable difficulty; may need assistance or walking aid
3 = unable to walk, or walks only with assistance and great effort

**17. Handwriting**

0 = normal
1 = some difficulty (e.g. slow, small letters); all words legible
2 = considerable difficulty; not all words legible; may need to use block letters
3 = majority of words are illegible

## C. Motor Complications

**18. Dyskinesias (presence)**

0 = absent
1 = present some of the time
2 = present a considerable part of the time
3 = present most or all of the time

**19. Dyskinesias (severity)**

0 = absent
1 = small amplitude
2 = moderate amplitude
3 = large amplitude

**20. Motor fluctuations (presence of 'off' periods)**

What proportion of the waking day is patient 'off' on average?

0 = none
1 = some of the time
2 = a considerable part of the time
3 = most or all of the time

**21. Motor fluctuations (severity of 'off' periods)**

0 = absent
1 = mild end-of-dose fluctuations
2 = moderate end-of-dose fluctuations; unpredictable fluctuations may occur occasionally
3 = severe end-of-dose fluctuations; unpredictable on-off oscillations occur frequently

Part A:

Part B:

Part C:

**TOTAL=___________________**

# CISI-PD

Instructions: At the end of the examination, mark the score better adjusted to your global impression of disease severity for each domain

**Motor signs**

0 Normal
1 Very mild
2 Mild
3 Mild to moderate
4 Moderate
5 Severe
6 Very severe

**Disability**

0 Normal
1 Minimal slowness and/or clumsiness
2 Slowness and/or clumsiness; no limitations
3 Limitation for demanding activities; does not need help for basic ADL
4 Limitation to perform basic ADL; help is required for some basic ADL
5 Great limitation to perform basic ADL; help is required for most or all basic ADL
6 Severely disabled; helpless; complete assistance needed

**Motor complications (dyskinesia and fluctuations)**

0 Not at all
1 Very mild
2 Mild
3 Mild to moderate
4 Moderate
5 Severe
6 Very severe

**Cognitive status**

0 Normal
1 Slowness and/or minimal cognitive problems
2 Mild cognitive problems; no limitations
3 Mild to moderate cognitive problems; does not need help for basic ADL
4 Moderate cognitive problems; help is required for some basic ADL
5 Severe cognitive problems; help is required for most or all basic ADL
6 Severely disabled; helpless; complete assistance needed

**TOTAL:_______________**

# NON-MOTOR SYMPTOM SCALE (NMSS)

**Severity: 0 = None**, **1 = Mild** (symptoms present but causes little distress or disturbance to patient), **2 = Moderate** (some distress or disturbance to patient), **3 = Severe** (major source of distress or disturbance to patient)
**Frequency**: **1 = Rarely** (<1/ week), **2 = Often** (1/week), **3 = Frequent** (several times per week), **4 = Very Frequent** (daily or all the time).

---

| | Severity 0-3 | Frequency 1-4 | Severity X Frequency |
|---|---|---|---|
| **Domain 1: Cardiovascular including falls** | | | |
| 1. Does the patient experience light-headedness, dizziness, weakness on standing from sitting or lying position? | ☐ | ☐ | ☐ |
| 2. Does the patient fall because of fainting or blacking out? | ☐ | ☐ | ☐ |
| **Domain 2: Sleep / Fatigue** | | | |
| 3. Does the patient doze off or fall asleep unintentionally during daytime activities (For example, during conversation, during mealtimes, or while watching television or reading)? | ☐ | ☐ | ☐ |
| 4. Does fatigue (tiredness) or lack of energy (not slowness) limit the patients daytime activities) | ☐ | ☐ | ☐ |
| 5. Does the patient have difficulties falling or staying asleep)? | ☐ | ☐ | ☐ |
| 6. Does the patient experience an urge to move the legs or restlessness in legs that improves with movements when he/she is sitting or lying down inactive? | ☐ | ☐ | ☐ |
| **Domain 3: Mood/ Cognition** | | | |
| 7. Has the patient lost interest in his/her surroundings? | ☐ | ☐ | ☐ |
| 8. Has the patient lost interest in doing things or lack motivation to start new activities? | ☐ | ☐ | ☐ |
| 9. Does the patient feel nervous, worried or frightened for no apparent reason? | ☐ | ☐ | ☐ |
| 10. Does the patient seem sad or depressed or has he/she reported such feelings? | ☐ | ☐ | ☐ |
| 11. Does the patient have flat moods without the normal ”highs“ and “lows“? | ☐ | ☐ | ☐ |
| 12. Does the patient have difficulty in experiencing pleasure from their usual activities or report that they lack pleasure? | ☐ | ☐ | ☐ |

| | Severity 0-3 | Frequency 1-4 | Severity X Frequency |
|---|---|---|---|
| **Domain 4: Perceptual problems / hallucinations** | | | |
| 13. Does the patient indicate that he/she sees things that are not there? | ☐ | ☐ | ☐ |
| 14. Does the patient have beliefs that you know are not true? (For example being harmed, being robbed or being unfaithful?) | ☐ | ☐ | ☐ |
| 15. Does the patient experience double vision? (2 separate real objects and not blurred vision) | ☐ | ☐ | ☐ |
| **Domain 5: Attention / Memory** | | | |
| 16. Does the patient have problems sustaining concentration during activities? | ☐ | ☐ | ☐ |
| 17. Does the patient forget things that he/she has been told a short time ago or events that happened in the last few days? | ☐ | ☐ | ☐ |
| 18. Does the patient forget to do things (for example, take tablets or turn off domestic appliances)? | ☐ | ☐ | ☐ |
| **Domain 6: Gastrointestinal tract** | | | |
| 19. Does the patient dribble salvia during the day? | ☐ | ☐ | ☐ |
| 20. Does the patient have difficulty swallowing? | ☐ | ☐ | ☐ |
| 21. Does the patient suffer from constipation? (Bowl action less than three times weekly?) | ☐ | ☐ | ☐ |
| **Domain 7: Urinary** | | | |
| 22. Does the patient have difficulty holding urine? (Urgency) | ☐ | ☐ | ☐ |
| 23. Does the patient have to void within two hours of last voiding? (Frequency) | ☐ | ☐ | ☐ |
| 24. Does the patient have to get up regularly at night to pass urine? (Nocturia) | ☐ | ☐ | ☐ |

| | Severity 0-3 | Frequency 1-4 | Severity X Frequency |
|---|---|---|---|
| **Domain 8: Sexual function** | | | |
| 25. Does the patient have altered interest in sex? (very much increased or decreased, please underline) | ☐ | ☐ | ☐ |
| 26. Does the patient have problems having sex? | ☐ | ☐ | ☐ |
| **Domain 9: Miscellaneous** | | | |
| 27. Does the patient suffer from pain not explained by other known conditions? (Is it related to intake of drugs and is it relieved by antiparkinson drugs?) | ☐ | ☐ | ☐ |
| 28. Does the patient report a change in ability to taste and smell? | ☐ | ☐ | ☐ |
| 29. Does the patient report a recent change in weight? (Not related to dieting) | ☐ | ☐ | ☐ |
| 30. Does the patient experience excessive sweating? (not related to hot weather) | ☐ | ☐ | ☐ |

**Total** =

# MINI-MENTAL STATE EXAMINATION (MMSE)

## 1. Orientation

| | | | |
|---|---|---|---|
| What is the year? | ☐ | Where are we now? (Country) | ☐ |
| What is the season? | ☐ | Where are we now? (City) | ☐ |
| What is the date? | ☐ | Where are we now? (Hospital) | ☐ |
| What is the month? | ☐ | Where are we now? (Floor) | ☐ |
| Where are we now? (State) | ☐ | | Score 0-9 |

## 2. Powers of retention

Ask the patient if you may test his memory.
Name three unrelated objects clearly and slowly (1 per second) **“lemon, key, ball”**.
Then ask the patient to name all three of them. The patient’s response is used for scoring (each word one point).
Repeat the words until the patient learns all of them, if possible. The patient has 5 attempts. It is important that he/she learns all of the words to prove the memory correctly.

☐☐☐ Score 0-3

## 3. Attention and calculation

“I would like you to count backward from 100 by sevens” (93, 86, 79, 72, 65, ….).
Stop after five answers and count the right ones.
Alternative: “Spell WORLD backwards” (D-L-R-O-W)
The higher score should be rated.

100 → 93 → 86 → 79 → 72 → 65

☐ ☐ ☐ ☐ ☐

WORLD → …………………………………….

☐ ☐ ☐ ☐ ☐

Score 0-5

## 4. Memory

“Earlier I told you the names of three things. Can you tell me what those were?”

☐☐☐ Score 0-3

**5. Naming**

Show the patient two simple objects, such as **wristwatch** and a **pencil**, and ask the patient to name them.

☐☐☐ Score 0-3

**6. Repetition**

"Repeat the phrase: '**No ifs, ands, or buts**'." (Only one attempt)

☐ Score 0-1

**7. Three-parted order**

Let the patient execute the order: **"Take the paper in your right hand, fold it in half, and put it on the floor".** (Give the patient a piece of blank paper). Give a point for every right order.

☐☐☐ Score 0-3

**8. Reacting**

Write on a blank piece of paper: **"Close your eyes"**. Ask the patient to read the text and follow the instruction. Give one point if he/she closes his/her eyes.

☐ Score 0-1

**9. Writing**

Give the patient a blank piece of paper: "**Make up and write a sentence about anything**". (This sentence must contain a noun and a verb, must make sense and must be written spontaneously).

☐ Score 0-1

**10. Reproducing**

Ask the patient to copy the figure. All 10 corners must be drawn and 2 must be overlapping to be scored. Trembling or twisting the figure is not important.

☐Score 0-1

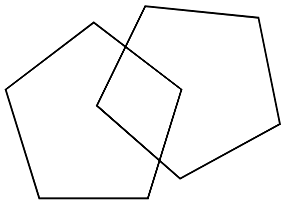

**Total=__________**

NAME :
Education : Date of birth :
Sex : DATE :

# MONTREAL COGNITIVE ASSESSMENT (MOCA)

Version 7.1 Original Version

## VISUOSPATIAL / EXECUTIVE

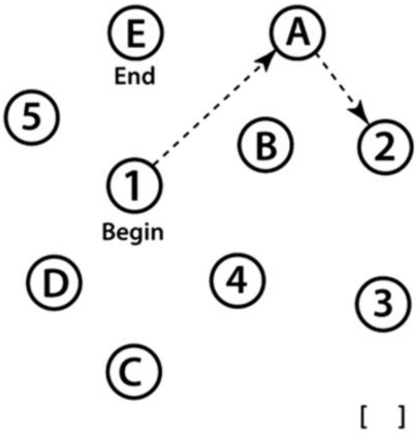


[ ]

Copy cube

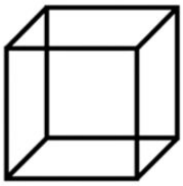

[ ]

Draw CLOCK (Ten past eleven)
( 3 points )

[ ] Contour [ ] Numbers [ ] Hands

POINTS: __/5

## NAMING

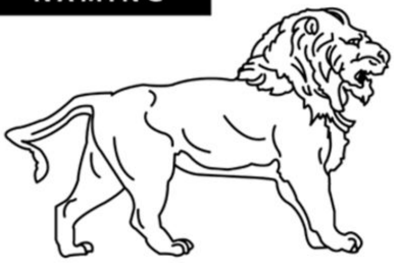

[ ]

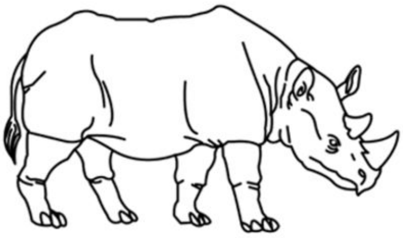

[ ]

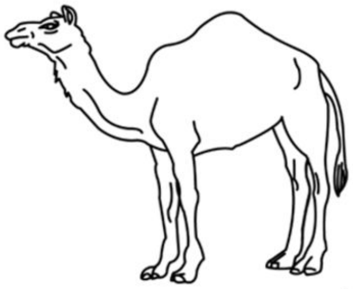

[ ]

__/3

## MEMORY

Read list of words, subject must repeat them. Do 2 trials, even if 1st trial is successful.
Do a recall after 5 minutes.

| | FACE | VELVET | CHURCH | DAISY | RED |
|---|---|---|---|---|---|
| 1st trial | | | | | |
| 2nd trial | | | | | |

No points

## ATTENTION

Read list of digits (1 digit/ sec.). Subject has to repeat them in the forward order [ ] 2 1 8 5 4
Subject has to repeat them in the backward order [ ] 7 4 2

__/2

Read list of letters. The subject must tap with his hand at each letter A. No points if ≥ 2 errors
[ ] F B A C M N A A J K L B A F A K D E A A A J A M O F A A B

__/1

Serial 7 subtraction starting at 100 [ ] 93 [ ] 86 [ ] 79 [ ] 72 [ ] 65
4 or 5 correct subtractions: **3 pts**, 2 or 3 correct: **2 pts**, 1 correct: **1 pt**, 0 correct: **0 pt**

__/3

## LANGUAGE

Repeat : I only know that John is the one to help today. [ ]
The cat always hid under the couch when dogs were in the room. [ ]

__/2

Fluency / Name maximum number of words in one minute that begin with the letter F [ ] _____ (N ≥ 11 words)

__/1

## ABSTRACTION

Similarity between e.g. banana - orange = fruit [ ] train – bicycle [ ] watch - ruler

__/2

## DELAYED RECALL

| | | FACE | VELVET | CHURCH | DAISY | RED | |
|---|---|---|---|---|---|---|---|
| DELAYED RECALL | Has to recall words **WITH NO CUE** | [ ] | [ ] | [ ] | [ ] | [ ] | Points for UNCUED recall only |
| **Optional** | Category cue | | | | | | |
| | Multiple choice cue | | | | | | |

__/5

## ORIENTATION

[ ] Date [ ] Month [ ] Year [ ] Day [ ] Place [ ] City

__/6

© Z.Nasreddine MD **www.mocatest.org** Normal ≥ 26 / 30

TOTAL __/30
Add 1 point if ≤ 12 yr edu

Administered by: ______________________________

# COGNITIVE ASSESSMENT

**Months reversed:**
**Ask the patient to give the months of the year backward, starting from December.**

2 or more mistakes: Cognition is abnormal

Abnormal ☐

Normal ☐

Number of mistakes _____

## WORD FLUENCY

Ask the patient to give words beginning with the letter B, M or S.
He/she should name as many words as possible during 1 minute.
(No repetitions, names or words from the same stem are allowed.)

| **F** | **A** | **S** | **Animals** |
|---|---|---|---|
| Correct answers___________ | Correct answers___________ | Correct answers___________ | Correct answers___________ |
| Repetitions_______________ | Repetitions_______________ | Repetitions_______________ | Repetitions _______________ |
| Mistakes_________________ | Mistakes_________________ | Mistakes_________________ | Mistakes_________________ |
