# Supplementary material for: Neurostimulation for Advanced Parkinson Disease and Quality of Life at 5 Years: A Nonrandomized Controlled Trial
Source: JAMA Netw Open. 2024 Jan 18;7(1):e2352177. doi: 10.1001/jamanetworkopen.2023.52177 (PMC10797423; doi:10.1001/jamanetworkopen.2023.52177)
Supplement: Supplement 2. — eTable 1. Outcomes at Baseline, 1-Year Follow-Up, and 5-Year Follow-Up in the Original Cohort eTable 2. Relative Changes and Effect Sizes for the Original Cohort at 1-Year and 5-Year Follow-Up eTable 3. Relative Changes and Effect Sizes for the Matched Cohort at 1-Year and 5-Year Follow-Up eFigure. Domains of Quality of Life and Motor Aspects in the Matched Cohort at Baseline, 1-, and 5-Year Follow-Up [file jamanetwopen-e2352177-s002.pdf]

Supplementary Online Content

Jost ST, Aloui S, Evans J, et al. Neurostimulation for advanced Parkinson disease and quality of life at 5 years: a nonrandomized controlled trial. *JAMA Netw Open*. 2024;7(1):e2352177. doi:10.1001/jamanetworkopen.2023.52177

eTable 1. Outcomes at Baseline, 1-Year Follow-Up, and 5-Year Follow-Up in the Original Cohort
eTable 2. Relative Changes and Effect Sizes for the Original Cohort at 1-Year and 5-Year Follow-Up
eTable 3. Relative Changes and Effect Sizes for the Matched Cohort at 1-Year and 5-Year Follow-Up
eFigure. Domains of Quality of Life and Motor Aspects in the Matched Cohort at Baseline, 1-, and 5-Year Follow-Up

This supplementary material has been provided by the authors to give readers additional information about their work.

© 2024 Jost ST et al. *JAMA Network Open.*

eTable 1. Outcomes at Baseline, 1-Year Follow-Up, and 5-Year Follow-Up in the Original Cohort

	STN-DBS														MED															
	Baseline			1-YFU			5-YFU			Baseline vs. 1-YFU[1]		Baseline vs. 5-YFU[1]		Baseline vs. 1-YFU vs. 5-YFU[2]	Baseline			1-YFU			5-YFU			Baseline vs. 1-YFU[1]		Baseline vs. 5-YFU[1]		Baseline vs. 1-YFU vs. 5-YFU[2]	STN-DBS vs. MED[3]	
	n	*Mean*	*SD*	*n*	*Mean*	*SD*	*n*	*Mean*	*SD*	*P*	*Δ [95% CI]*	*P*	*Δ [95% CI]*	*P*	*n*	*Mean*	*SD*	*n*	*Mean*	*SD*	*n*	*Mean*	*SD*	*P*	*Δ [95% CI]*	*P*	*Δ [95% CI]*	*P*	*P*	*Δ [95% CI]*
**PDQ-8 Summary Index**	60	31.8	14.5	61	23.4	14.6	57	37.1	17.0	<.001*	8.2 [4.7; 11.6]	.03*	-5.2 1 [-9.7; -0.6]	<.001*	46	21.3	15.5	42	21.7	15.3	44	29.4	18.7	.95	0.1 [-4.8; 5.1]	.005*	-7.7 [-13.0; -2.5]	.004*	.46	-2.6 [-9.4; 4.3]
Mobility	60	1.9	1.0	61	1.2	1.1	59	1.8	1.2	<.001*	0.7 [0.3; 1.0]	.67	0.1 [-0.3; 0.4]	<.001*	46	1.1	1.3	42	1.2	1.3	44	1.2	1.3	.66	-0.1 [-0.5; 0.3]	.46	-0.1 [-0.5; 0.2]	.74	.40	-0.1 [-0.7; 0.4]
Activities of daily living	60	1.4	1.1	61	0.9	1.2	59	1.5	1.2	.003*	0.5 [0.2; 0.7]	.42	-0.1 [-0.5; 0.2]	<.001*	46	1.1	1.3	42	1.0	1.2	44	1.4	1.3	.74	0.1 [-0.4; 0.5]	.20	-0.3 [-0.7; 0.2]	.19	.63	-0.2 [-0.7; 0.3]
Emotional well-being	60	1.1	0.9	61	0.9	0.9	59	1.5	1.1	.08	0.2 [0.0; 0.4]	.01*	-0.4 [-0.7; -0.1]	<.001*	46	0.8	1.1	42	0.7	0.9	44	1.2	1.1	.47	0.1 [-0.2; 0.5]	.06	-0.4 [-0.7; 0.0]	.03*	.87	0.0 [-0.4; 0.5]
Social support	60	1.0	0.9	61	0.7	0.8	59	1.4	1.0	.04*	0.3 [0.0; 0.5]	.02*	-0.4 [-0.7; -0.1]	<.001*	46	0.5	1.0	42	0.3	0.6	44	0.9	0.9	.13	0.2 [-0.1; 0.6]	.07	-0.4 [-0.8; 0.0]	.006*	.88	0.0 [-0.5; 0.6]
Cognition	60	1.3	0.9	61	1.1	1.0	59	1.7	0.9	.12	0.2 [-0.1; 0.5]	.09	-0.3 [-0.6; 0.0]	.006*	46	0.7	1.0	42	0.8	1.1	44	1.2	1.2	.45	-0.1 [-0.5; 0.2]	.01*	-0.5 [-0.8; -0.1]	.05	.48	-0.2 [-0.7; 0.3]
Communication	60	1.1	0.9	61	1.0	0.9	57	1.6	1.1	.79	0.0 [-0.2; 0.3]	<.001*	-0.6 [-0.9; -0.3]	<.001*	46	0.9	0.9	42	0.7	0.8	44	1.1	1.0	.15	0.2 [-0.1; 0.5]	.17	-0.2 [-0.6; 0.1]	.04*	.13	0.3 [-0.1; 0.8]
Bodily discomfort	60	1.6	1.2	61	1.1	1.2	59	1.6	1.1	.004*	0.5 [0.2; 0.8]	.85	0.0 [-0.3; 0.4]	.009*	46	1.0	1.2	42	1.3	1.2	44	1.4	1.2	.24	-0.2 [-0.6; 0.2]	.07	-0.4 [-0.8; 0.0]	.26	.14	-0.4 [-0.9; 0.1]
Stigma	60	0.9	1.0	61	0.6	0.9	58	0.9	1.1	.02*	0.3 [0.0; 0.5]	.73	0.1 [-0.3; 0.4]	.09	46	0.7	1.0	42	0.8	1.1	44	1.0	1.2	.72	-0.1 [-0.5; 0.3]	.15	-0.3 [-0.6; 0.1]	.40	.17	-0.3 [-0.8; 0.1]
**UPDRS-Motor examination**	61	32.0	12.8	58	25.7	10.8	41	31.0	14.2	<.001*	6.1 [3.0; 9.2]	.38	1.9 [-2.4; 6.1]	<.001*	46	29.1	13.2	42	29.1	12.8	44	35.6	15.2	.70	-0.7 [-4.2; 2.9]	<.001*	-6.5 [-10.2; -2.9]	.004*	.003*	-0.2 [-0.7; 0.3]
**SCOPA-M total**	61	22.8	8.4	58	16.7	7.7	41	21.1	10.1	<.001*	5.9 [4.1; 7.8]	.23	1.7 [-1.1; 4.4]	<.001*	46	19.5	8.5	42	17.5	9.0	45	23.7	9.9	.23	1.4 [-0.9; 3.7]	<.001*	-4.6 [-6.5; -2.6]	<.001*	<.001*	-6.2 [-9.5; -2.9]
SCOPA-M activities of daily living	61	7.1	3.3	61	5.7	3.4	61	7.8	4.1	.002*	1.4 [0.5; 2.2]	.18	-0.7 [-1.8; 0.3]	<.001*	46	6.3	3.0	42	5.5	3.7	43	7.8	4.2	.11	0.6 [-0.2; -1.4]	.001*	-1.7 [-2.6; -0.7]	<.001*	.23	-0.9 [-2.4; 0.6]
SCOPA-M motor Complications (Md; IQR)	61	(4.0)	(2.0; 7.0)	61	(2.0)	(0.0; 3.5)	61	(2.0)	(0.0; 4.5)	<.001*	-2.0 [-2.5; -1.0]	<.001*	-1.5 [-2.5; -1.0]	<.001*	46	(2.0)	(2.0; 4.0)	42	(2.0)	(0.0; 3.0)	45	(4.0)	(2.0; 4.5)	.001*	-1.0 [-1.5; -0.5]	.34	0.0 [-0.5; 1.0]	.004*	<.001*	-2.0 [-3.0; -1.0]
**LEDD**	62	1147.9	531.6	61	512.9	314.1	61	743.8	496.8	<.001*	646.9 [512.1; 781.8]	<.001*	423.7 [-267.3; 580.1]	<.001*	46	694.7	383.4	42	876.8	444.8	41	1028.6	450.9	<.001*	-180.8 [-277.7; -83.9]	<.001*	-345.9 [-463.4; -228.4]	<.001*	<.001*	-769.6 [-980.2; -558.9]

Legend: Outcomes at baseline, 1-year follow-up, and 5-year follow-up for the STN-DBS and MED groups. Significant results are highlighted in bold font. Multiple comparisons due to multiple outcome parameters were corrected with Benjamini-Hochberg's method. Post-hoc, we explored PDQ-8 domains and within-group changes between different visits.

[1] Wilcoxon signed rank test or paired t-tests between baseline and follow-up to analyze within-group changes of outcome parameters.
[2] Friedman test or repeated-measures ANOVA when parametric test criteria were fulfilled.
[3] Mann-Whitney U tests or repeated measures ANOVA were used to explore between-group differences of change scores (baseline vs. 5-year follow-up) between STN-DBS and MED group.

Abbreviations: 1-YFU = 1-year follow-up; **5-YFU** = 5-year follow-up; **IQR** = interquartile range; **LEDD** = levodopa equivalent daily dose; **MED** = standard-of-care medical treatment; **Md** = Median; **PDQ-8** = 8-item Parkinson's Disease Questionnaire; **SCOPA-M** = Scales for Outcomes in Parkinson's disease-motor scale; **STN-DBS** = subthalamic nucleus deep brain stimulation; **UPDRS** = Unified Parkinson's Disease Rating Scale.

© 2024 Jost ST et al. *JAMA Network Open.*

eTable 2. Relative Changes and Effect Sizes for the Original Cohort at 1-Year and 5-Year Follow-Up

	Within-group changes								Between-group differences at 5-year follow-up (Classification)
	Baseline to 1-year follow-up				Baseline to 5-year follow-up				
	Relative change (%)		Effect size (Classification)		Relative change (%)		Effect size (Classification)		Effect size favoring STN-DBS
	STN-DBS	MED	STN-DBS	MED	STN-DBS	MED	STN-DBS	MED	
**PDQ-8 Summary Index**	26.4	-1.9	0.58 (moderate)	0.03	-16.7	-38.0	0.37 (small)	0.52 (moderate)	0.19
**UPDRS-Motor examination**	19.7	0.0	0.49 (small)	0.00	3.1	-22.3	0.08	0.49 (small)	0.57 (moderate)
**SCOPA-M total**	26.8	10.3	0.73 (moderate)	0.24 (small)	7.5	-21.5	0.20 (small)	0.49 (small)	0.69 (moderate)
SCOPA-M activities of daily living	19.7	12.7	0.42 (small)	0.27 (small)	-9.9	-23.8	0.21 (small)	0.50 (moderate)	0.25 (small)
SCOPA-M motor complications	47.7	36.7	0.75 (moderate)	0.61 (moderate)	40.9	-10.0	0.64 (moderate)	0.17	0.89 (large)
**LEDD**	55.3	-26.2	1.19 (large)	0.47 (small)	35.5	-48.0	0.76 (moderate)	0.87 (large)	1.55 (large)

Legend: Relative changes and effect sizes from baseline to 1-year follow up and baseline to 5-year follow-up in the STN-DBS and MED group. Effect sizes of the between-group comparison STN-DBS vs. MED at 5-year follow-up.

Relative change = (mean $test_{baseline}$ − mean $test_{follow\text{-}up}$)/ mean $test_{baseline}$ x 100
Cohen's effect size = (mean $test_{baseline}$ − mean $test_{follow\text{-}up}$)/ SD $test_{baseline}$
Cohen's *d* = (mean pre-post $change_{treatment\ group}$ –mean pre-post $change_{control\ group}$) / SD $pretest_{pooled\ groups}$
Cohen's *d* can be classified as 'small' (0.20≥*d*<0.50), 'moderate' (0.50≥*d*<0.80) and 'large' (*d*≥0.80)

Abbreviations: LEDD = levodopa equivalent daily dose; **MED** = standard-of-care medical treatment; **PDQ-8** = 8-item Parkinson's Disease Questionnaire; **SCOPA-M =** Scales for Outcomes in Parkinson's disease-motor scale; **STN-DBS** = subthalamic nucleus deep brain stimulation; **UPDRS** = Unified Parkinson's Disease Rating Scale.

© 2024 Jost ST et al. *JAMA Network Open.*

eTable 3. Relative Changes and Effect Sizes for the Matched Cohort at 1-Year and 5-Year Follow-Up

	Within-group changes								Between-group differences at 5-year follow-up (Classification)
	Baseline to 1-year follow-up				Baseline to 5-year follow-up				
	Relative change (%)		Effect size (Classification)		Relative change (%)		Effect size (Classification)		
	STN-DBS	MED	STN-DBS	MED	STN-DBS	MED	STN-DBS	MED	Effect size favoring STN-DBS
**PDQ-8 Summary Index**	25.5	-5.0	0.52 (moderate)	0.08	-10.3	-49.4	0.21 (small)	0.77 (moderate)	0.57 (moderate)
**UPDRS-Motor examination**	7.7	3.2	0.20 (small)	0.07	0.2	-13.8	0.01	0.30 (small)	0.31 (small)
**SCOPA-M total**	19.2	9.5	0.48 (small)	0.22 (small)	6.39	-19.5	0.16	0.44 (small)	0.60 (moderate)
SCOPA-M activities of daily living	26.1	6.0	0.50 (moderate)	0.13	-11.6	-25.4	0.22 (small)	0.55 (moderate)	0.26 (small)
SCOPA-M motor complications	52.9	33.3	0.67 (moderate)	0.60 (moderate)	47.1	-27.3	0.59 (moderate)	0.56 (moderate)	1.11 (large)
**LEDD**	62.2	-17.0	1.38 (large)	0.45 (small)	47.0	-35.4	1.04 (large)	0.33 (small)	1.95 (large)

Legend: Relative changes and effect sizes from baseline to 1-year follow up and baseline to 5-year follow-up in the STN-DBS and MED group. Effect sizes of the between-group comparison STN-DBS vs. MED at 5-year follow-up.

Relative change = (mean $test_{baseline}$ − mean $test_{follow\text{-}up}$)/ mean $test_{baseline}$ x 100
Cohen's effect size = (mean $test_{baseline}$ − mean $test_{follow\text{-}up}$)/ SD $test_{baseline}$
Cohen's effect size for differences in change scores between the STN-DBS and MED group= (mean pre-post $change_{treatment\ group}$ − mean pre-post $change_{control\ group}$) / SD $pretest_{pooled\ groups}$
Cohen's *d* can be classified as 'small' (0.20≥*d*<0.50), 'moderate' (0.50≥*d*<0.80) and 'large' (*d*≥0.80).

Abbreviations: LEDD = levodopa equivalent daily dose; **MED** = standard-of-care medical treatment; **PDQ-8** = 8-item Parkinson's Disease Questionnaire; **SCOPA-M** = Scales for Outcomes in Parkinson's disease-motor scale; **STN-DBS** = subthalamic nucleus deep brain stimulation; **UPDRS** = Unified Parkinson's Disease Rating Scale.

© 2024 Jost ST et al. *JAMA Network Open.*

eFigure. Domains of Quality of Life and Motor Aspects in the Matched Cohort at Baseline, 1-, and 5-Year Follow-Up

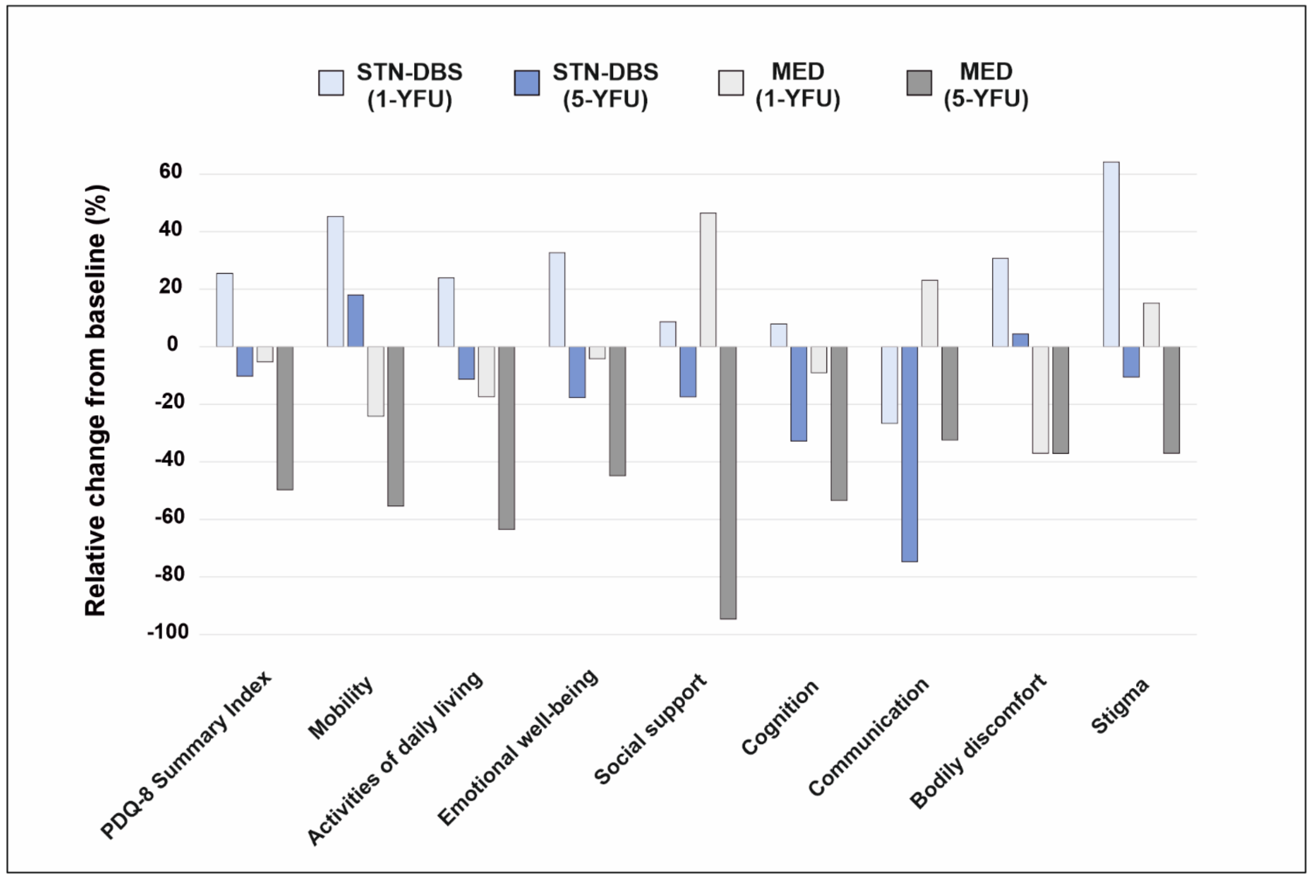

Legend: Change in the 8-item Parkinson's Disease Questionnaire (PDQ-8) Summary Index and subscores from baseline to 1- and 5-year follow-up. Positive scores indicate improvement and negative scores indicate worsening. Significant longitudinal within-group changes between the three visits are highlighted with a black star.
Abbreviations: 1-YFU = 1-year follow-up; **5-YFU** = 5-year follow-up**; MED** = standard-of-care medical treatment; **PDQ-8 =** 8-item Parkinson's Disease Questionnaire; **STN-DBS** = subthalamic nucleus deep brain stimulation.

© 2024 Jost ST et al. *JAMA Network Open.*
