# Supplementary material for: Neurostimulation for Advanced Parkinson Disease and Quality of Life at 5 Years: A Nonrandomized Controlled Trial
Source: JAMA Netw Open. 2024 Jan 18;7(1):e2352177. doi: 10.1001/jamanetworkopen.2023.52177 (PMC10797423; doi:10.1001/jamanetworkopen.2023.52177)
Supplement: Supplement 3. — Nonauthor Collaborators [file jamanetwopen-e2352177-s003.pdf]

Supplemental Online Content: Nonauthor Collaborators
*First name, last name, and suffix (if applicable) are required and will appear in PubMed.

***Group Name(s): EUROPAR and the International Parkinson and Movement Disorders Society Non-Motor Parkinson's Disease Study Group**

***First Name and Middle Initial(s)**	***Last Name**	***Suffix (eg, Jr, III)**	Academic Degrees	Institution	Location (city, state/province, country)	Role or Contribution, eg, chair, principal investigator	Group (if more than 1 Group listed in the byline) and/or Subgroup (eg, Steering Committee)
Pablo	Martinez-Martin			National Centre of Epidemiology and CIBERNED, Carlos III Institute of Health	Madrid, Spain	Investigator	EUROPAR
Carmen	Rodriguez-Blazquez			National Centre of Epidemiology and CIBERNED, Carlos III Institute of Health	Madrid, Spain	Investigator	EUROPAR
Ray	Chaudhuri			Kings College Hospital	London, United Kingdom	Chair	EUROPAR
Alexandra	Rizos			Kings College Hospital	London, United Kingdom	Investigator	EUROPAR
Anna	Sauerbier			Kings College Hospital	London, United Kingdom	Investigator	EUROPAR
Miriam	Parry			Kings College Hospital	London, United Kingdom	Investigator	EUROPAR
Dhaval	Trivedi			Kings College Hospital	London, United Kingdom	Investigator	EUROPAR
Davide	Martino			Kings College London	London, United Kingdom	Investigator	EUROPAR
Per	Odin			Department of Neurology, Lund University Hospital	Lund, Sweden	Investigator	EUROPAR
Angelo	Antonini			Department for Parkinson’s Disease, IRCCS San Camillo	Venice, Italy	Investigator	EUROPAR
Fabrizio	Stocchi			Department of Neurology, IRCCS San Raffaele	Rome, Italy	Investigator	EUROPAR
Jacobus J	van Hilten			Department of Neurology, Leiden University Medical Center	Leiden, The Netherlands	Investigator	EUROPAR
Teus	van Laar			University of Groningen	Groningen, The Netherlands	Investigator	EUROPAR
Cristian	Falup-Pecurariu			Department of Neurology, Faculty of Medicine, Transilvania University	Braşov, Romania	Investigator	EUROPAR
Espen	Dietrichs			Oslo University Hospital	Oslo, Norway	Investigator	EUROPAR
Kelly E.	Lyons			University of Kansas Medical Center	Kansas City, Kansas, US	Investigator	EUROPAR
Dan	Weintraub			Hospital of the University of PA	Philadelphia, USA	Investigator	EUROPAR

*First name, last name, and suffix (if applicable) are required and will appear in PubMed.

***First Name and Middle Initial(s)**	***Last Name**	***Suffix (eg, Jr, III)**	Academic Degrees	Institution	Location (city, state/province, country)	Role or Contribution, eg, chair, principal investigator	Group (if more than 1 Group listed in the byline) and/or Subgroup (eg, Steering Committee)
Monica	Kurtis			Department of Neurology. Hospital Ruber Internacional	Madrid, Spain	Investigator	EUROPAR
Marcos	Serrano-Dueñas			Movement Disorder and Biostatistic Units, Neurological Service, Carlos Andrade Marín Hospital	Quito, Ecuador	Investigator	EUROPAR
Vanderci	Borges			Movement Disorders Section, Department of Neurology, Universidade Federal de São Paulo	São Paulo, Brazil	Investigator	EUROPAR
Madhuri	Behari			Department of Neurology, All India Institute of Medical Sciences	New Delhi, India	Investigator	EUROPAR
Kalyan	Bhattacharya			RG Kar Medical College and Institute of Neuroscience	Kolkata, India	Investigator	EUROPAR
Hrishikesh	Kumar			RG Kar Medical College and Institute of Neuroscience	Kolkata, India	Investigator	EUROPAR
Bhim	Singhal			Bombay Hospital Institute of Medical Sciences	Mumbai, India	Investigator	EUROPAR
Roongroj	Bhidayasiri			Chulalongkorn University Hospital	Bangkok, Thailand	Investigator	EUROPAR
Lim	Shen-Yang			University of Malaya	Kualalampur, Malaysia	Investigator	EUROPAR
Marisol	Gallardo			Hospital Dr. Domingo Luciani	Caracas, Venezuela	Investigator	EUROPAR
Kazuo	Abe			Hyogo Medical College	Nishinomiya, Japan	Investigator	EUROPAR
Sevasti	Bostantjopoulo u			Third Department of Neurology, Aristotle University of Thessaloniki	Thessaloniki, Greece	Investigator	EUROPAR
Jose	Martin Rabey			Department of Neurology, Assaf Harofeh Medical Center, Tel Aviv University	Zerifin, Israel	Investigator	EUROPAR
Federico	Micheli			Parkinson's Disease and Other Movement Disorders Unit, Hospital de Clinicas Jose de San Martin	Buenos Aires, Argentina	Investigator	EUROPAR

Supplemental Online Content: Nonauthor Collaborators
*First name, last name, and suffix (if applicable) are required and will appear in PubMed.

***First Name and Middle Initial(s)**	***Last Name**	***Suffix (eg, Jr, III)**	Academic Degrees	Institution	Location (city, state/province, country)	Role or Contribution, eg, chair, principal investigator	Group (if more than 1 Group listed in the byline) and/or Subgroup (eg, Steering Committee)
Mayela	Rodriguez-Violante			Movement Disorders Unit, Instituto Nacional de Neurologia y Neurocirugía	Mexico DF, Mexico	Investigator	EUROPAR
Giulio	Riboldazzi			Macchi Foundation Varese and Department of Rehabilitation, Le Terrazze Hospital	Cunardo, Italy	Investigator	EUROPAR
Maria Jose	Catalan			Hospital Clinico San Carlos	Madrid, Spain	Investigator	EUROPAR
Cathy	Ellis			Medway Maritime Hospital	Kent, United Kingdom	Investigator	EUROPAR
Lorna	Bean			Medway Maritime Hospital	Kent, United Kingdom	Investigator	EUROPAR
Belinda	Kessel			Princess Royal University Hospital, King’s College Hospital	Orpington, United Kingdom	Investigator	EUROPAR
Paul	Worth			Department of Neurology, Norfolk and Norwich University Hospital	Cambridge, United Kingdom	Investigator	EUROPAR
Gemma	Shearing			Department of Neurology, Norfolk and Norwich University Hospital	Cambridge, United Kingdom	Investigator	EUROPAR
Rani	Sophia			Yeovil Hospital NHS Foundation Trust	Somerset, United Kingdom	Investigator	EUROPAR
Jagdish	Sharma			United Lincolnshire Hospitals NHS Trust	Lincoln, United Kingdom	Investigator	EUROPAR
Monty	Silverdale			Salford Royal NHS Foundation Trust	Manchester, United Kingdom	Investigator	EUROPAR
Suvankar	Pal			Forth Valley Royal Hospital	Edinburgh, United Kingdom	Investigator	EUROPAR
Keyoumars	Ashkan			King’s College Hospital	London, United Kingdom	Investigator	EUROPAR
Charles	Adler			The Parkinson's Disease and Moveme	Arizona, USA	Investigator	International Parkinson and Movement Disorders Society Non-Motor Parkinson's Disease Study Group

Supplemental Online Content: Nonauthor Collaborators
*First name, last name, and suffix (if applicable) are required and will appear in PubMed.

***First Name and Middle Initial(s)**	***Last Name**	***Suffix (eg, Jr, III)**	Academic Degrees	Institution	Location (city, state/province, country)	Role or Contribution, eg, chair, principal investigator	Group (if more than 1 Group listed in the byline) and/or Subgroup (eg, Steering Committee)
Paolo	Barone			Center for Neurodegenerative Diseas	Salerno, Italy	Investigator	International Parkinson and Movement Disorders Society Non-Motor Parkinson's Disease Study Group
David J.	Brooks			Institute of Neuroscience, Newcastle	Newcastle, UK, and Aarhus	Investigator	International Parkinson and Movement Disorders Society Non-Motor Parkinson's Disease Study Group
Richard	Brown			Department of Medicine, University o	Birmingham, United Kingdo	Investigator	International Parkinson and Movement Disorders Society Non-Motor Parkinson's Disease Study Group
Marc	Cantillon			Reviva Pharmaceuticals, Inc.	Santa Clara, CA, USA	Investigator	International Parkinson and Movement Disorders Society Non-Motor Parkinson's Disease Study Group
Camille	Carroll			Faculty of Medicine and Dentistry, Ur	Plymouth, UK	Investigator	International Parkinson and Movement Disorders Society Non-Motor Parkinson's Disease Study Group
Miguel	Coelho			FAS Center for Systems Biology, Harv	Cambridge, MA, USA	Investigator	International Parkinson and Movement Disorders Society Non-Motor Parkinson's Disease Study Group

Supplemental Online Content: Nonauthor Collaborators
*First name, last name, and suffix (if applicable) are required and will appear in PubMed.

***First Name and Middle Initial(s)**	***Last Name**	***Suffix (eg, Jr, III)**	Academic Degrees	Institution	Location (city, state/province, country)	Role or Contribution, eg, chair, principal investigator	Group (if more than 1 Group listed in the byline) and/or Subgroup (eg, Steering Committee)
Haidar S.	Dafsari			Department of Neurology, University	Cologne, Germany	Principal Investigator	International Parkinson and Movement Disorders Society Non-Motor Parkinson's Disease Study Group
Tove	Henriksen			Movement Disorder Clinic, University	Copenhagen, NV, Denmark	Investigator	International Parkinson and Movement Disorders Society Non-Motor Parkinson's Disease Study Group
Michele	Hu			Oxford Parkinson's Disease Centre, U	University of Oxford, UK	Investigator	International Parkinson and Movement Disorders Society Non-Motor Parkinson's Disease Study Group
Peter	Jenner			Neurodegenerative Diseases Researc	London, UK	Investigator	International Parkinson and Movement Disorders Society Non-Motor Parkinson's Disease Study Group
Milica	Kramberger			Division of Clinical Geriatrics, Departr	Stockholm, Sweden, and Lj	Investigator	International Parkinson and Movement Disorders Society Non-Motor Parkinson's Disease Study Group
Padma	Kumar			Parkinson's Disease Service for the O	Newcastle, NSW, Australia	Investigator	International Parkinson and Movement Disorders Society Non-Motor Parkinson's Disease Study Group

Supplemental Online Content: Nonauthor Collaborators
*First name, last name, and suffix (if applicable) are required and will appear in PubMed.

***First Name and Middle Initial(s)**	***Last Name**	***Suffix (eg, Jr, III)**	Academic Degrees	Institution	Location (city, state/province, country)	Role or Contribution, eg, chair, principal investigator	Group (if more than 1 Group listed in the byline) and/or Subgroup (eg, Steering Committee)
Mónica	Kurtis			Functional Movement Disorders Unit	Madrid, Spain	Investigator	International Parkinson and Movement Disorders Society Non-Motor Parkinson's Disease Study Group
Simon	Lewis			Brain and Mind Centre, University of	Sydney, NSW, Australia	Investigator	International Parkinson and Movement Disorders Society Non-Motor Parkinson's Disease Study Group
Irene	Litvan			Department of Neurosciences Mover	San Diego, USA	Investigator	International Parkinson and Movement Disorders Society Non-Motor Parkinson's Disease Study Group
Kelly	Lyons			University of Kansas Medical Center	Kansas City, KS, USA	Investigator	International Parkinson and Movement Disorders Society Non-Motor Parkinson's Disease Study Group
Davide	Martino			Department of Clinical Neuroscience:	Calgary, Canada	Investigator	International Parkinson and Movement Disorders Society Non-Motor Parkinson's Disease Study Group
Mario	Masellis			Hurvitz Brain Sciences Program, Sunr	Toronto, ON, Canada	Investigator	International Parkinson and Movement Disorders Society Non-Motor Parkinson's Disease Study Group

Supplemental Online Content: Nonauthor Collaborators
*First name, last name, and suffix (if applicable) are required and will appear in PubMed.

***First Name and Middle Initial(s)**	***Last Name**	***Suffix (eg, Jr, III)**	Academic Degrees	Institution	Location (city, state/province, country)	Role or Contribution, eg, chair, principal investigator	Group (if more than 1 Group listed in the byline) and/or Subgroup (eg, Steering Committee)
Hideki	Mochizuki			Department of Neurology, Osaka Uni	Osaka, Japan	Investigator	International Parkinson and Movement Disorders Society Non-Motor Parkinson's Disease Study Group
James F.	Morley			Parkinson Disease Research, Educatic	Philadelphia, PA, USA	Investigator	International Parkinson and Movement Disorders Society Non-Motor Parkinson's Disease Study Group
Melissa	Nirenberg			Department of Neurology, NYU Schoc	New York, NY, USA	Investigator	International Parkinson and Movement Disorders Society Non-Motor Parkinson's Disease Study Group
Javier	Pagonabarraga			Movement Disorders Unit, Sant Pau H	Barcelona, Spain	Investigator	International Parkinson and Movement Disorders Society Non-Motor Parkinson's Disease Study Group
Jalesh	Panicker			Neurology, National Hospital for Neu	London, United Kingdom	Investigator	International Parkinson and Movement Disorders Society Non-Motor Parkinson's Disease Study Group
Nicola	Pavese			Newcastle Magnetic Resonance Cent	Newcastle upon Tyne, Unit	Investigator	International Parkinson and Movement Disorders Society Non-Motor Parkinson's Disease Study Group

Supplemental Online Content: Nonauthor Collaborators
*First name, last name, and suffix (if applicable) are required and will appear in PubMed.

***First Name and Middle Initial(s)**	***Last Name**	***Suffix (eg, Jr, III)**	Academic Degrees	Institution	Location (city, state/province, country)	Role or Contribution, eg, chair, principal investigator	Group (if more than 1 Group listed in the byline) and/or Subgroup (eg, Steering Committee)
Eero	Pekkonen			Department of Neurology, Helsinki U	Helsinki, Finland	Investigator	International Parkinson and Movement Disorders Society Non-Motor Parkinson's Disease Study Group
Ron	Postuma			Research Institute of McGill Universit	Montréal, Canada	Investigator	International Parkinson and Movement Disorders Society Non-Motor Parkinson's Disease Study Group
Raymond	Rosales			Department of Neurology and Psychi	Manila , Philippines	Investigator	International Parkinson and Movement Disorders Society Non-Motor Parkinson's Disease Study Group
Anthony	Schapira			Department of Clinical Neuroscience	London, UK	Investigator	International Parkinson and Movement Disorders Society Non-Motor Parkinson's Disease Study Group
Tanya	Simuni			Department of Neurology , Northwes	Chicago , IL , USA	Investigator	International Parkinson and Movement Disorders Society Non-Motor Parkinson's Disease Study Group
Fabrizio	Stocchi			University and Institute for Research	Rome, Italy	Investigator	International Parkinson and Movement Disorders Society Non-Motor Parkinson's Disease Study Group

Supplemental Online Content: Nonauthor Collaborators
*First name, last name, and suffix (if applicable) are required and will appear in PubMed.

***First Name and Middle Initial(s)**	***Last Name**	***Suffix (eg, Jr, III)**	Academic Degrees	Institution	Location (city, state/province, country)	Role or Contribution, eg, chair, principal investigator	Group (if more than 1 Group listed in the byline) and/or Subgroup (eg, Steering Committee)
Indu	Subramanian			UCLA/West LA VA	Los Angeles, CA, USA	Investigator	International Parkinson and Movement Disorders Society Non-Motor Parkinson's Disease Study Group
Michele	Tagliati			Cedars-Sinai Medical Center	Los Angeles, CA, USA	Investigator	International Parkinson and Movement Disorders Society Non-Motor Parkinson's Disease Study Group
Lars	Timmermann			Department of Neurology, University	Marburg, Germany	Investigator	International Parkinson and Movement Disorders Society Non-Motor Parkinson's Disease Study Group
Michele	Tinazzi			Department of Neuroscience, Biomed	Verona, Italy	Investigator	International Parkinson and Movement Disorders Society Non-Motor Parkinson's Disease Study Group
Jon	Toledo			Department of Pathology & Laborato	Houston, TX, USA	Investigator	International Parkinson and Movement Disorders Society Non-Motor Parkinson's Disease Study Group
Yoshio	Tsuboi			Department of Neurology, Fukuoka U	Fukuoka, Japan	Investigator	International Parkinson and Movement Disorders Society Non-Motor Parkinson's Disease Study Group

Supplemental Online Content: Nonauthor Collaborators
*First name, last name, and suffix (if applicable) are required and will appear in PubMed.

***First Name and Middle Initial(s)**	***Last Name**	***Suffix (eg, Jr, III)**	Academic Degrees	Institution	Location (city, state/province, country)	Role or Contribution, eg, chair, principal investigator	Group (if more than 1 Group listed in the byline) and/or Subgroup (eg, Steering Committee)
Richard	Walker			Northumbria Healthcare NHS Founda	Tyne and Wear, United Kin	Investigator	International Parkinson and Movement Disorders Society Non-Motor Parkinson's Disease Study Group
