# Supplementary material for: Neurostimulation for Advanced Parkinson Disease and Quality of Life at 5 Years: A Nonrandomized Controlled Trial
Source: JAMA Netw Open. 2024 Jan 18;7(1):e2352177. doi: 10.1001/jamanetworkopen.2023.52177 (PMC10797423; doi:10.1001/jamanetworkopen.2023.52177)
Supplement: Supplement 4. — Data Sharing Statement [file jamanetwopen-e2352177-s004.pdf]

Data Sharing Statement

Jost. Neurostimulation for Advanced Parkinson Disease and Quality of Life at 5 Years. *JAMA Netw Open*. Published January 18, 2024. doi:10.1001/jamanetworkopen.2023.52177

Data
Data available: No

Additional Information
Explanation for why data not available: The data used to support this study's findings are available from the corresponding authors upon reasonable request.
